# AlgicideDB: a comprehensive database enhanced by large language models for algicide management and discovery

**DOI:** 10.3389/fmicb.2025.1611403

**Published:** 2025-06-18

**Authors:** Zhangqi Zuo, Jing Hu, Chaowei Zhang, Zuoqi Wang, Lei Chen, Fei Li, Xi Xiao

**Affiliations:** ^1^Ocean College, Zhejiang University, Zhoushan, Zhejiang, China; ^2^College of Agriculture and Biotechnology, Zhejiang University, Hangzhou, China; ^3^State Key Laboratory of Marine Pollution, City University of Hong Kong, Kowloon, Hong Kong SAR, China

**Keywords:** harmful algal blooms, algicide, aquatic toxicity, large language model, retrieval augmented generation

## Abstract

Harmful algal blooms (HABs) are increasing in frequency and intensity worldwide, posing significant threats to aquatic ecosystems, fisheries, and human health. While chemical algicides are widely used for HABs control due to their rapid efficacy, the lack of systematic data integration and concerns over environmental toxicity limit their broader application. To address these challenges, we developed AlgicideDB, a manually curated database containing 1,672 algicidal records on 542 algicides targeting 110 algal species. Using this database, we analyzed the physicochemical properties of algicides and proposed an algicide-likeness scoring function to facilitate the exploration of compounds with antialgal properties. Additionally, we evaluated the acute toxicity of algicidal compounds to non-target aquatic organisms of different trophic levels to assess their ecological risks. The platform also incorporates a large language model (LLM) enhanced by retrieval-augmented generation (RAG) to address HAB-related queries, supporting decision-making and facilitating knowledge dissemination. AlgicideDB, available at http://algicidedb.ocean-meta.com/#/, serves as an innovative and comprehensive platform to explore algicidal compounds and facilitate the development of safe and effective HAB control strategies.

## 1 Introduction

Algae play a crucial role in aquatic ecosystems as primary producers, supporting food webs and maintaining ecological balance (Field et al., 1998; Lizotte, 2001; Woodward, 2007). However, some algal species have the potential to undergo uncontrolled proliferation in favorable conditions, resulting in the formation of mass blooms and/or a high production of phycotoxins, known as harmful algal blooms (HABs) (Anderson et al., 2012). Excessive algal growth generates dense biomass that forms unsightly scums and foam, blocks sunlight to phytoplankton and benthic floras, depletes oxygen during decay, and disrupts ecosystems, fisheries, and recreational activities (Anderson et al., 2012; Brown et al., 2020). Moreover, certain algae contribute to biofilm formation, thus fouling water intake structures and impacting equipment maintenance and water quality in public water systems and industrial cooling water systems (Lv et al., 2022; Valeriani et al., 2024). Additionally, some HAB-forming species release harmful toxins that accumulate through the food web posing severe health risks to humans and other organisms (Yan et al., 2022). Over the past few decades, the frequency and intensity of HABs have increased globally (Dai et al., 2023), driven by factors such as eutrophication, global warming, and anthropogenic activities (Xiao et al., 2019). To address these challenges, it is essential to develop effective and environmentally friendly strategies to control algal outbreaks and mitigate their associated risks.

Currently, strategies for preventing or managing HABs involve physical methods such as nutrient load reduction and hydrodynamic regulation, chemical interventions like algicide application, and biological strategies including predation and competitive exclusion (Gallardo-Rodríguez et al., 2019). Among these approaches, chemical algicides are often used in emergencies due to their rapid and effective suppression of algal blooms (Huisman et al., 2018). Previous efforts have identified numerous effective algicides, including synthetic chemicals, natural products derived from plants and microorganisms, and their structural analogs (Zhu et al., 2021). However, these data are scattered across the literature and lack comprehensive risk assessments of environmental toxicity, which hinders data accessibility and practical application. Several publicly available databases, such as the US EPA's ECOTOX database and the NCBI's PubChem BioAssay, contain a vast amount of ecotoxicological information, including some data related to algal toxicity. However, within these broad resources, information relevant to algicides is often dispersed among extensive datasets covering diverse organisms and endpoints. This lack of a focused collection and specialized tools makes it challenging to systematically extract, analyze, and apply this information specifically for algicide research and development. There is an urgent need to develop a comprehensive database that organizes algicide-related information and facilitates the evaluation of aquatic toxicity. The integration of information also creates opportunities for developing domain-specific large language models (LLM) to support algicide research and HABs management.

Here, we present AlgicideDB (http://algicidedb.ocean-meta.com/#/), a manually curated database that integrates comprehensive data on algicides. Distinguished from existing resources, AlgicideDB offers structured data organization through its specialized focus on algicides, enhanced usability with specifically designed tools, and unique functionalities like algicidal-likeness scoring and aquatic toxicity predictions. Currently, the database contains 1,672 records of algicidal activity, 1,329 algicide-algae pairs, 542 unique algicides, and 110 algal species. On this basis, we conducted a thorough analysis of these data, developed algicidal-likeness scoring based on the molecular properties of algicides, and performed aquatic toxicity predictions to assess their ecological risks. Additionally, the platform features LLM-based question-answering service designed to response HAB-related queries. Overall, AlgicideDB serves as an integrated platform for algicide discovery, management, and the development of effective HABs control strategies.

## 2 Materials and methods

### 2.1 Data collection and curation

**Figure 1:**
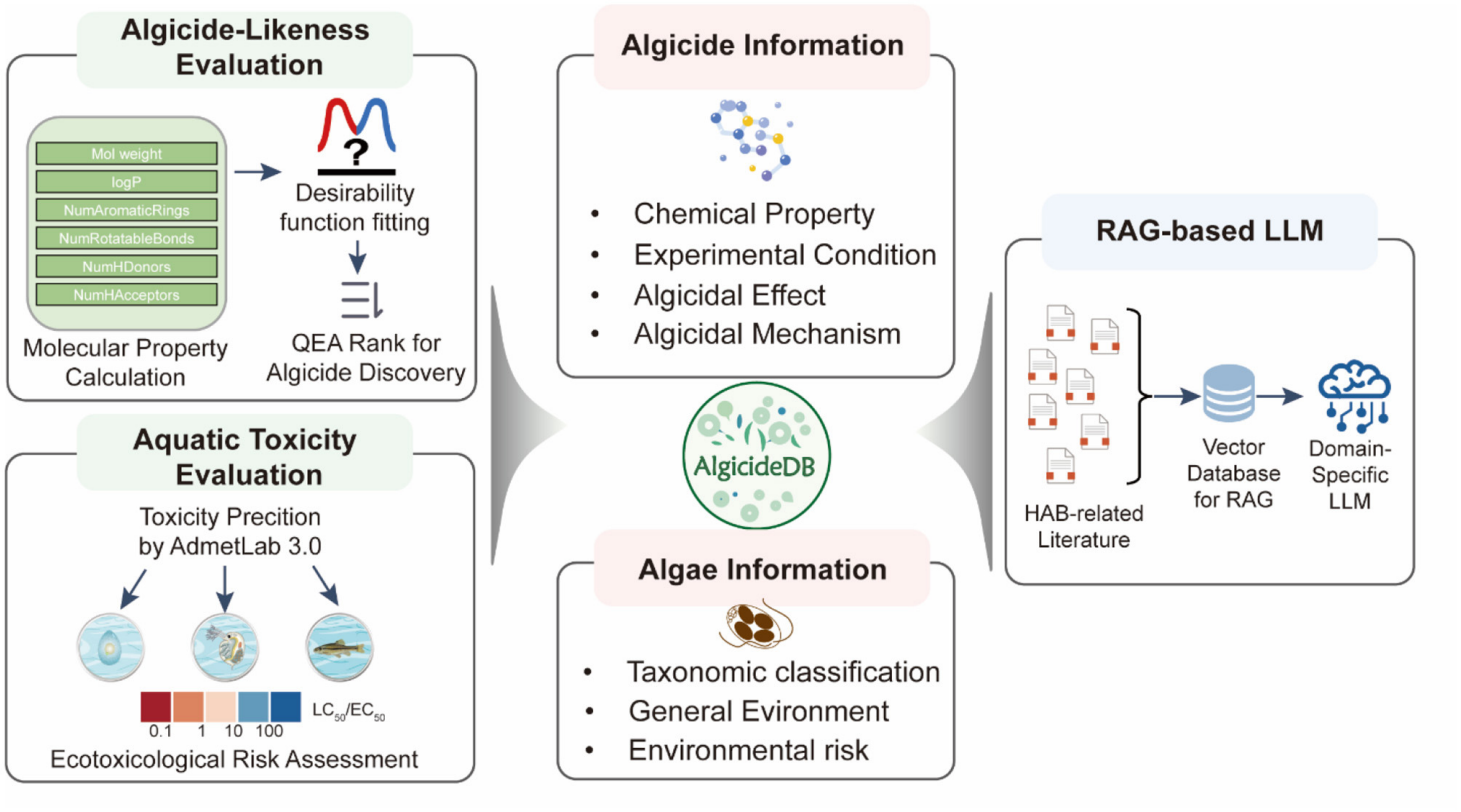
Workflow for the construction of AlgicideDB, including algicide and algae information collection, algicide-likeness scoring, aquatic toxicity assessment, and the development of RAG-based LLM.

We searched the Web of Science and PubMed databases using keywords such as “algicidal,” “antialgal,” “algal bloom control,” and “harmful algae mitigation.” From the retrieved literature, records of algicidal activity were extracted, including information on target algae, algicides, algicidal effects, experimental conditions, and the mechanisms (Figure 1). Details of these 204 articles are provided in the Supporting Information file. Additional information on target algae, such as their classifications and general environment, was supplemented using the AlgaeBase (Guiry and Guiry, 2024) (https://www.algaebase.org/). The phylogeny was constructed based on taxonomic classification data from AlgaeBase for each algal species in our database, which established a hierarchical tree structure representing taxonomic relationships, and was then visualized using the ggtree v3.5.3 package (Yu et al., 2017). Details of algicides, including their form, source, and structural information, were curated from the literature and further analyzed using NPClassifier (Kim et al., 2021) and ClassyFire (Djoumbou Feunang et al., 2016) tools for chemical classification. To organize the curated data, we created five interconnected database tables (Supplementary Figure S1) to store information on algae species, algae strains, algicides, algicidal activity records, and literature references. The data processing workflow involved extracting experimental records from literature, collecting additional information on algae from AlgaeBase and chemical information from the literature and PubChem. Finally, all collected data was systematically checked for consistency and completeness to ensure data quality.

### 2.2 Quantitative estimation of algicide-likeness

The concept of quantitative estimate of drug-likeness (QED), introduced by Bickerton et al. (2012), provides a scoring system to evaluate drug-likeness by describing the distribution of molecular descriptors, with scores ranging from zero (low drug-likeness) to one (high drug-likeness). Based this concept, Sorin et al. developed the quantitative estimate of pesticide-likeness (QEP) to assess pesticide potential (Avram et al., 2014). Inspired by these approaches, we established a quantitative estimate of algicide-likeness (QEA) to evaluate the algicidal potential of compounds. First, six molecular descriptors were calculated using RDKit (Landrum, 2006) including molecular weight (MW), octanol-water partition coefficient (logP), the number of hydrogen bond acceptors (HBA), the number of hydrogen bond donors (HBD), the number of rotatable bonds (nRotB), and the number of aromatic rings (nArR) (Avram et al., 2014). The selection of these descriptors was guided by principles established in drug and agrochemical discovery (Bickerton et al., 2012; Avram et al., 2014). For each descriptor, a desirability function was fitted (Equation 1), with values ranging from 0 to 1, representing the likelihood of a molecule being an algicide (Supplementary Figure S2). The coefficients (o, a, b, and c) used in the desirability function were derived from the distribution of physicochemical properties, as detailed in Supplementary Table S1. For the continuous descriptors (MW and logP), the optimal bin size for creating these frequency counts was determined using Shimazaki-Shinimoto histogram binning method (Shimazaki and Shinomoto, 2007). For the discrete descriptors (HBA, HBD, nRotB, and nArR), the frequency of each integer value was directly counted. The resulting frequency counts and the corresponding values for each molecular descriptor then served as input for curve fitting, which was processed using the https://findcurves.com/ Curve Fitting and Surface Fitting Web Site. Finally, the individual desirability functions, *d*f(*i*), for each molecular descriptor were combined into the QEA score using geometric means and logarithms, as described in Equation 2. We calculated the algicide-likeness scores for algicides in the database (positive set) and 2,000 randomly selected compounds from the ChEMBL dataset (negative set). A receiver operating characteristic (ROC) curve was generated based on these scores, and the area under the curve (AUC) was computed to evaluate the performance of the QEA in distinguishing algicides from non-algicides.


(1) $$\begin{array}{l} df=o+a^{*}e^{-e^{\frac{-x-b}{c}}-\;\frac{x-b}{c}+\;1} \end{array}$$

(2) $$\begin{array}{l} QEA=\;e^{\frac{1}{n}\;\sum_{i=1}^{i=n}\ln\left(dfi\right)},\;d\left(i\right)>0;QEA=0,\;df\left(i\right)\;\leq 0 \end{array}$$

### 2.3 Aquatic toxicity evaluation of algicide

The simplified molecular input line entry specification (SMILES) representations of the algicides were obtained using the Dicimer tool (Rajan et al., 2023) and the PubChem database (https://pubchem.ncbi.nlm.nih.gov/). These SMILES were input into the ADMET Evaluation functionality of ADMETlab 3.0 (Fu et al., 2024) to predict toxicity values for three aquatic species: *Tetrahymena pyriformis* (protozoa), *Daphnia magna* (zooplankton), and *Pimephales promelas* (fish). The predictions included comprehensive toxicity endpoints under standardized experimental conditions, i.e., the 48 h median effective concentration (EC_50, 48h_) for *T. pyriformis* (50% growth inhibition), the 48 h median lethal concentration (LC_50, 48h_) for *D. magna* (50% lethality), and the 96 h median lethal concentration (LC_50, 96h_) for *P. promelas* (50% lethality). According to the guidelines established by the US Environmental Protection Agency (EPA) (US EPA, 2015), the toxicity levels were defined as follows: practically non-toxic (LC_50_ or EC_50_ > 100 mg/L), slightly toxic (10~100 mg/L), moderately toxic (1~10 mg/L), highly toxic (0.1~1 mg/L), and very highly toxic (<0.1 mg/L).

### 2.4 Construction of RAG-based LLM for HAB-related queries

**Figure 2:**
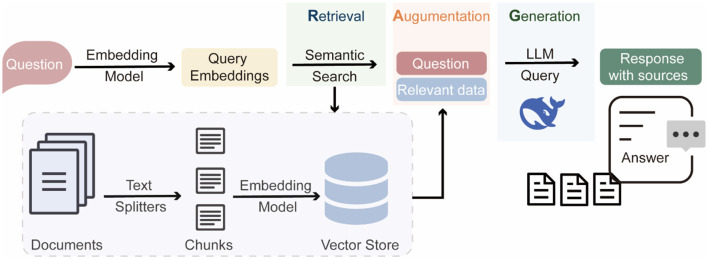
Workflow of RAG-based LLM agent system.

We constructed a knowledge base using RAGFlow (https://github.com/infiniflow/ragflow), an open-source Retrieval-Augmented Generation (RAG) engine (Figure 2). A total of 208 review articles on HABs published between 2010 and 2025 were manually collected. These articles cover topics related to the formation mechanisms, ecological impacts, monitoring methods, and management strategies of HABs. The collected articles were split by RAGFlow, resulting in 14,954 semantically meaningful text chunks. These chunks were then vectorized with the embedding model BAAI/bge-base-en-v1.5 (https://huggingface.co/BAAI/bge-base-en-v1.5) and stored in the retrieval database. This retrieval database was further integrated with the LLM (DeepSeek-v3), enabling retrieval-augmented generation for HABs-related question answering. To evaluate the performance of the RAG-based LLM, we used DeepSeek to automatically generate 1,040 high-quality question-answer pairs, where questions were derived from randomly selected literature chunks. The generated questions were then used as input for the RAG-based LLM to produce responses. We collected the generated answers and the retrieved references, and evaluated their quality using metrics (Supplementary Table S2) provided by Ragas (https://github.com/explodinggradients/ragas).

### 2.5 Database implementation and functionalities

The AlgicideDB platform is hosted on an Nginx (v.1.16.1) web server (http://nginx.org/) running on a CentOS 7.4.1,708 system. The backend is built using a SQLite database (https://www.sqlite.org/) for efficient data management, and developed with the Django (v.4.2.8) framework (https://www.djangoproject.com/). The frontend is built with Vue (v. 3.5.13) JavaScript framework (https://v3.vuejs.org/) to provide interactive user interface. The platform integrates functionalities including searching algicide records, uploading and downloading data, calculating algicide-likeness scores, predicting aquatic toxicity values. Additionally, a RAG-based LLM chatbot is embedded to offer a question-and-answer service, enabling real-time responses to HAB-related queries.

## 3 Results

### 3.1 Diversity and characteristics of algae targeted by algicides

**Figure 3:**
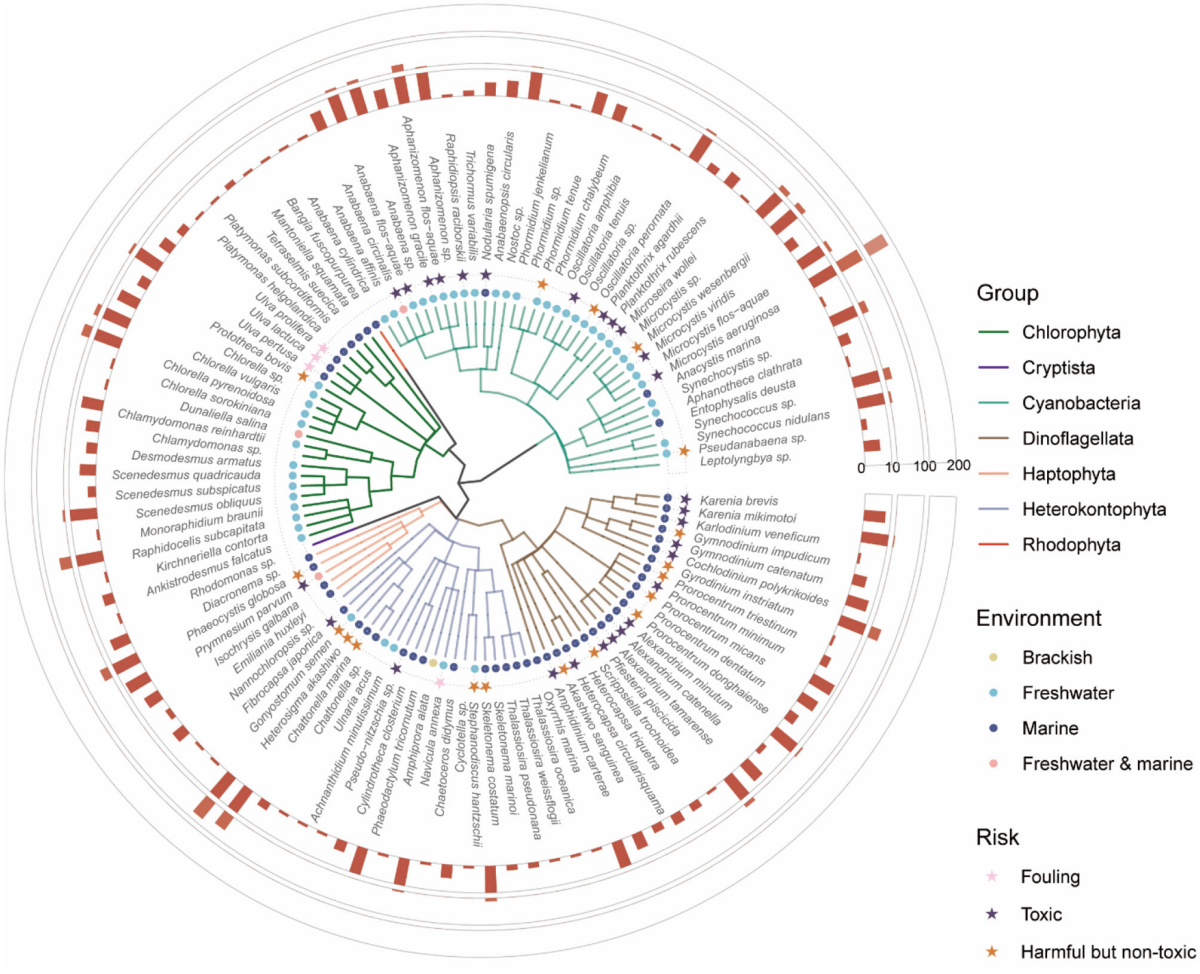
Phylogenetic and ecological diversity of algae targeted by algicides. Different colored branches correspond to different phyla. The colored dots denote the environmental distribution of each species: brackish (yellow), freshwater (cyan), marine (blue), and freshwater and marine (pink). Stars represent the risk profiles of each species, classified as fouling (pink), toxic (purple), and harmful but non-toxic (orange). The outermost bar chart illustrates the number of unique algicides associated with each species.

In total, our database includes 110 algal species and 246 algal strains, with the majority belonging to the Cyanobacteria (37 species), *Chlorophyta* (23 species), *Dinoflagellata* (22 species), and Heterokontophyta (21 species) (Figure 3). Cyanobacteria and dinoflagellates are prominent targets due to their toxic and harmful nature, which poses significant challenges in ecological management. Notably, *Microcystis aeruginosa* (Cyanobacteria), linked to 260 algicides, along with harmful species such as *Heterosigma akashiwo* (Heterokontophyta), associated with 88 algicides, and *Cochlodinium polykrikoides* (*Dinoflagellata*), related to 50 algicides, are extensively studied. In contrast, some widely distributed green algae and diatoms are commonly used as controls to evaluate the selectivity of algicides, including species such as *Chlorella pyrenoidosa* (*Chlorophyta*), *Raphidocelis subcapitata* (*Chlorophyta*) and *Phaeodactylum tricornutum* (Heterokontophyta). Moreover, green macroalgae such as *Ulva* are well-studied for their association with biofouling and the formation of green tides, large-scale blooms that cause significant environmental and economic impacts. The distribution of algal species in the database highlights current research priorities, with a focus on managing harmful and toxic species while exploring the selectivity of algicides for broader ecological applications.

### 3.2 Chemical profiles and sources of algicides

**Figure 4:**
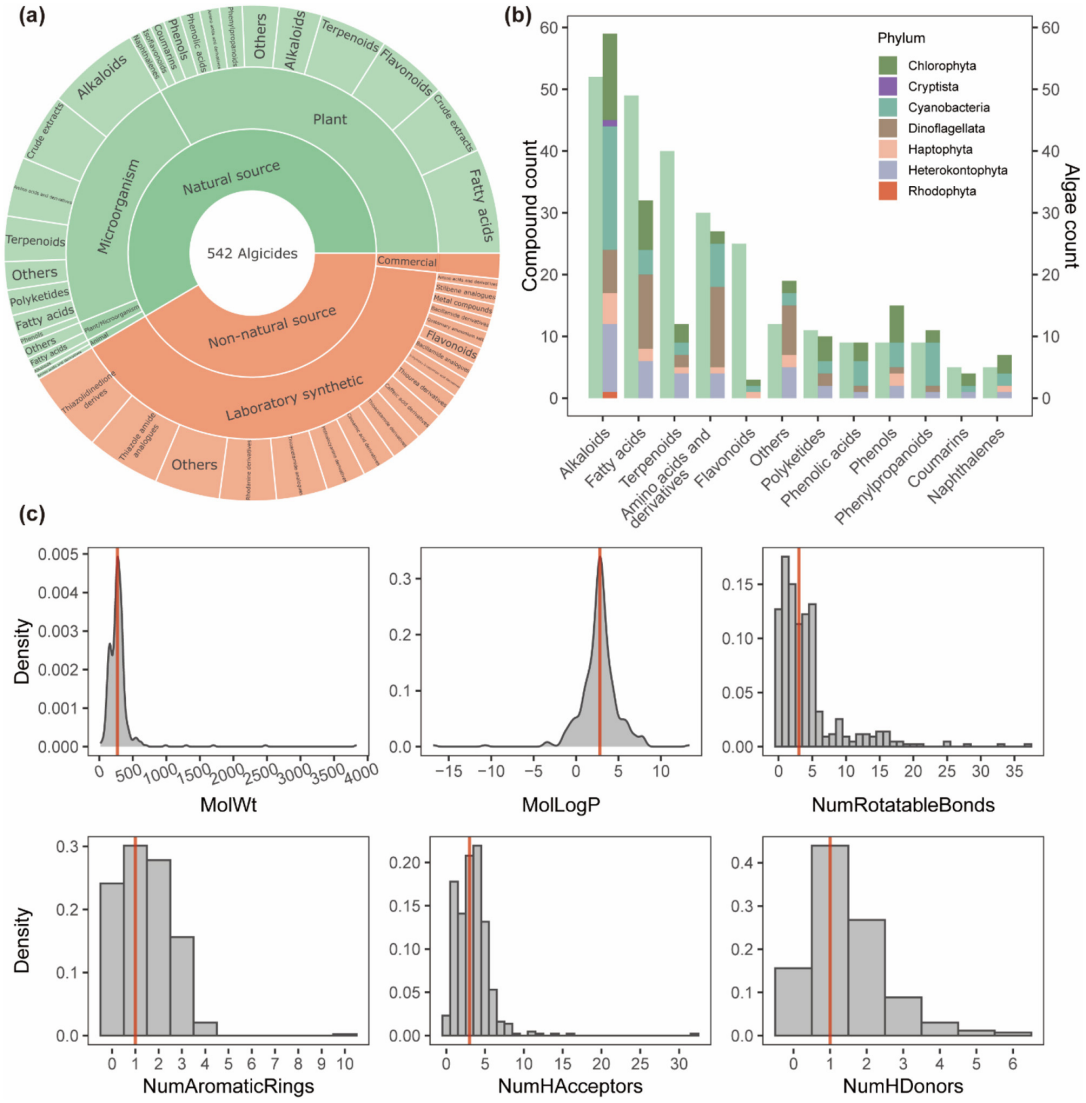
Chemical profiles and sources of algicides in AlgicideDB. **(a)** Distribution of 542 algicides based on their sources, including natural (plants, microorganisms, and animals) and non-natural origins (laboratory synthetic and commercial products). **(b)** Number of algicides across different chemical classes derived from natural sources (left bars) and the number of algal taxa targeted by each class (right bars). The colors in the right bars represent the distribution of algal phyla. **(c)** Molecular property distributions of algicides including molecular weight (MolWt), octanol-water partition coefficient (MolLogP), number of rotatable bonds, number of aromatic rings, number of hydrogen bond acceptors, and number of hydrogen bond donors. The red lines indicate median values for each descriptor.

The AlgicideDB database includes a total of 542 algicides, of which 58.5% are derived from natural sources and 41.5% are classified as non-natural sources (Figure 4a). Natural sources include a diverse range of plants, microorganisms, animals (summarized in Supplementary Tables S3–S5), showing the important role of natural products in algicide discovery. Common examples include aquatic plants such as *Myriophyllum spicatum* (Zhu et al., 2010) and *Arundo donax* (giant reeds) (Hong et al., 2010), macroalgae like *Sargassum* (Bazes et al., 2009; Cho, 2013), terrestrial plant materials such as barley straw (Murray et al., 2010; Xiao et al., 2014) and rice hulls (Park et al., 2009), and marine bacteria including *Vibrio sp*. (Wang et al., 2012; Li et al., 2014), and *Bacillus sp*. (Zhao et al., 2014; Quan et al., 2021).

The most abundant natural product classes are alkaloids, fatty acids, and terpenoids (Figure 4b), which exhibit broad-spectrum activity against diverse algal taxa. Especially, alkaloids in the database target all seven algal phyla represented, highlighting their effectiveness as algicidal agents. Non-natural sources also contribute significantly to the diversity of the database, with numerous synthetic derivatives and analogs inspired by natural compounds (Supplementary Figure S3; Supplementary Table S6), and commercial algicides (Supplementary Table S7). These products expand the chemical diversity of available algicides, offering enhanced efficacy and environmental adaptability. Besides, our analysis of the 61 algicide-algae pairs with known mechanisms reveals mechanistic diversity across different chemical classes. Photosynthesis inhibition and membrane disruption were the most prevalent mechanisms, each accounting for 25.3% of the entries, followed closely by oxidative stress induction (20.5%), and metabolic inhibition (6.0%). Fatty acids primarily disrupted photosynthesis (40.0%) and cell membranes (20.0%), while alkaloids tended to induce oxidative stress (37.5%) and membrane disruption (31.2%). Notably, the mechanisms of action for over 90% of the algicide-algae pairs in AlgicideDB remain to be determined, indicating a significant area for future investigation.

### 3.3 Algicide property analysis and algicide-likeness evaluation

We analyzed six key molecular descriptors for the algicides in the database: molecular weight (MW), octanol-water partition coefficient (logP), number of rotatable bonds, number of aromatic rings, number of hydrogen bond acceptors (HBA), and number of hydrogen bond donors (HBD). These properties are crucial for evaluating pesticide-like molecules and their environmental behavior (Avram et al., 2014). The distributions of these descriptors (Figure 4c) indicate that the MW of algicides ranges from approximately 100 to 1,000 g/mol, with the majority falling between 200 and 500 g/mol. The logP distribution shows a peak around 2–5, reflecting moderate hydrophobicity favorable to algicidal activity. Most compounds have fewer than five rotatable bonds, ensuring a balance between molecular flexibility and stability. Additionally, the majority of algicides have 1–2 aromatic rings and fewer than five HBA and two HBD.

**Figure 5:**
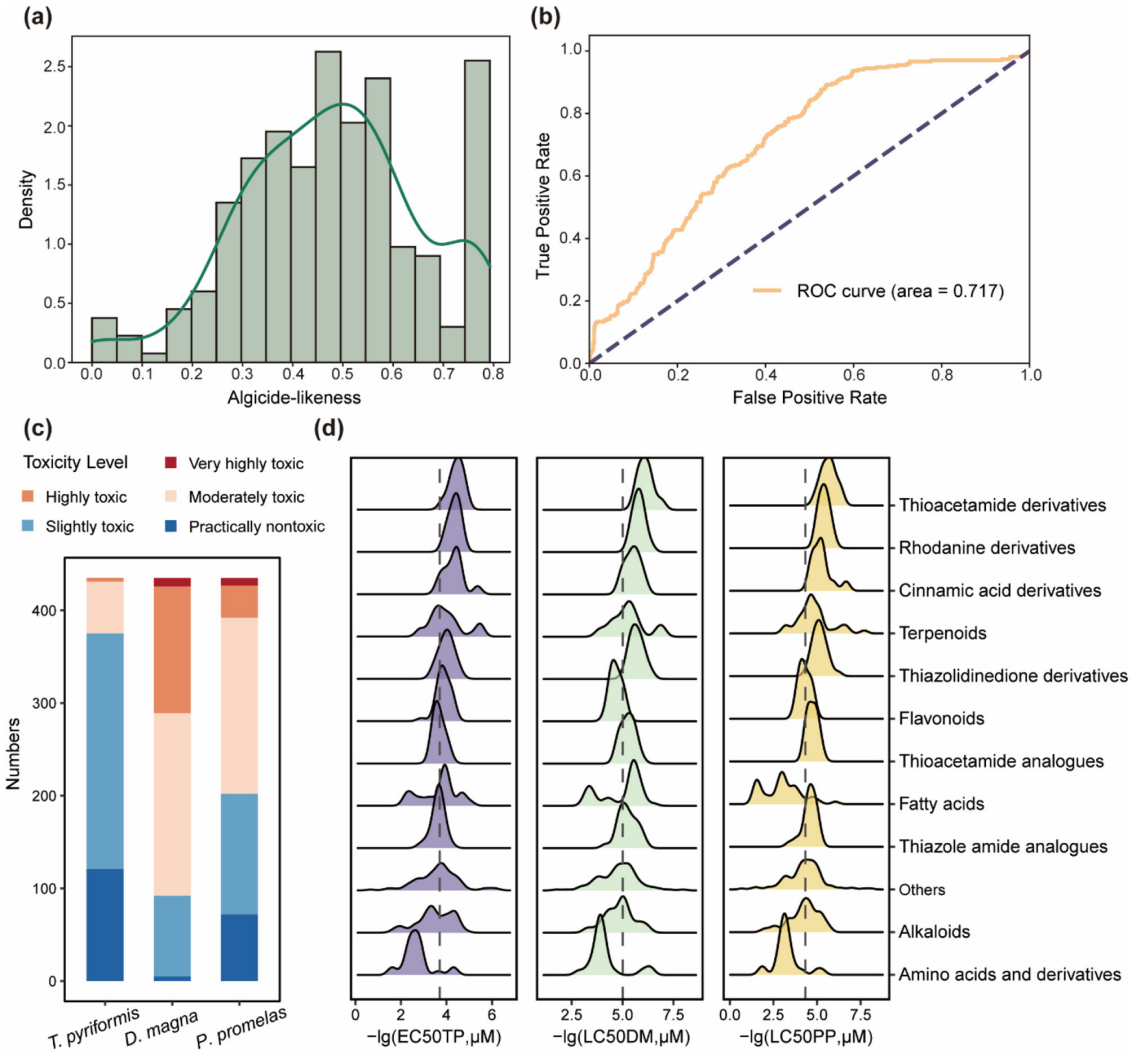
Algicide-likeness distribution and ecotoxicological risk assessment of algicides. **(a)** Distribution of algicide-likeness scores. **(b)** ROC curve for the algicide-likeness scoring function, with an area under the curve (AUC) of 0.717. **(c)** Predicted toxicity levels of algicides for three aquatic species: *Tetrahymena pyriformis, Daphnia magna*, and *Pimephales promelas*, categorized into five levels (practically nontoxic, slightly toxic, moderately toxic, highly toxic, and very highly toxic). **(d)** Toxicity distribution of chemical classes, represented as –lg(EC50TP) for *T. pyriformis*, –lg(LC50DM) for *D. magna*, and -lg(LC50PP) for *P. promelas*. Larger values indicate higher toxicity.

Using these molecular descriptors, we developed a desirability function to quantify algicide-likeness. The algicide-likeness scores were computed (Figure 5a), and their ability to distinguish algicides from non-algicides was evaluated using a negative set of 2,000 randomly selected compounds from the ChEMBL dataset. The receiver operating characteristic (ROC) analysis yielded an area under the curve (AUC) of 0.717 (Figure 5b), demonstrating that the algicide-likeness metric effectively differentiates algicides, which aligns with the performance observed in similar quantitative likeness estimation methods within the broader agrochemical domain (e.g., herbicide-likeness (QEH) AUC = 0.721, insecticide-likeness (QEI) AUC = 0.668, and fungicide-likeness (QEF) AUC = 0.677) (Avram et al., 2014). The algicide-likeness evaluation has been integrated into our web-based platform to facilitate high-throughput screening and algicide discovery.

### 3.4 Ecotoxicological risk assessment of algicides

Besides algicide-likeness evaluation, we also considered the potential toxicity of algicides to non-target aquatic organisms. Among the 204 studies we collected, only 20 have evaluated the toxicological effects of algicides on non-algal aquatic species. To address this limitation, we predicted the toxicity of algicides across three trophic levels: *T. pyriformis* (protozoa), *D. magna* (zooplankton), and *P. promelas* (fish) (Figure 5c). The predictions indicate that algicides generally exhibit relatively lower toxicity toward *T. pyriformis*. However, more than half of the compounds were categorized as moderately toxic or higher for *D. magna* and *P. promelas*. Further analysis of toxicity levels across chemical classifications revealed that amino acid-derived algicides show relatively lower toxicity to all three aquatic species, suggesting their potential as environmentally friendly algicides (Figure 5d). This lower toxicity may be attributed to their natural occurrence in ecosystems, which enables safer interactions with aquatic organisms, as well as their potential for safe environmental degradation. Conversely, certain classes, such as thiazolidinedione and rhodanine derivatives, were associated with higher toxicity levels across multiple species, possibly due to their distinctive sulfur-containing heterocyclic structures. These findings underscore the importance of incorporating toxicity assessments into the development of safer and more sustainable algicides. Considering that the confidence of these predictions may vary across different chemical scaffolds, especially for novel structures underrepresented in toxicological databases, experimental validation remains essential to confirm these toxicity patterns.

### 3.5 Application and performance of RAG-based LLM

To explore innovative approaches in HABs management, we applied a RAG-based LLM to construct a domain-specific knowledge base for answering HABs-related queries. When a user submits a question, the system first retrieves relevant literature chunks from the database, which were then combined with the user's query as input for the LLM to enhance the contextuality and factuality of the generated response. The replies not only address the query but also provide references to the retrieved literature as supporting evidence (Supplementary Figure S4). This RAG-based LLM has been integrated into the AlgicideDB platform to provide users with support for HABs-related research and decision-making in HABs management.

**Figure 6:**
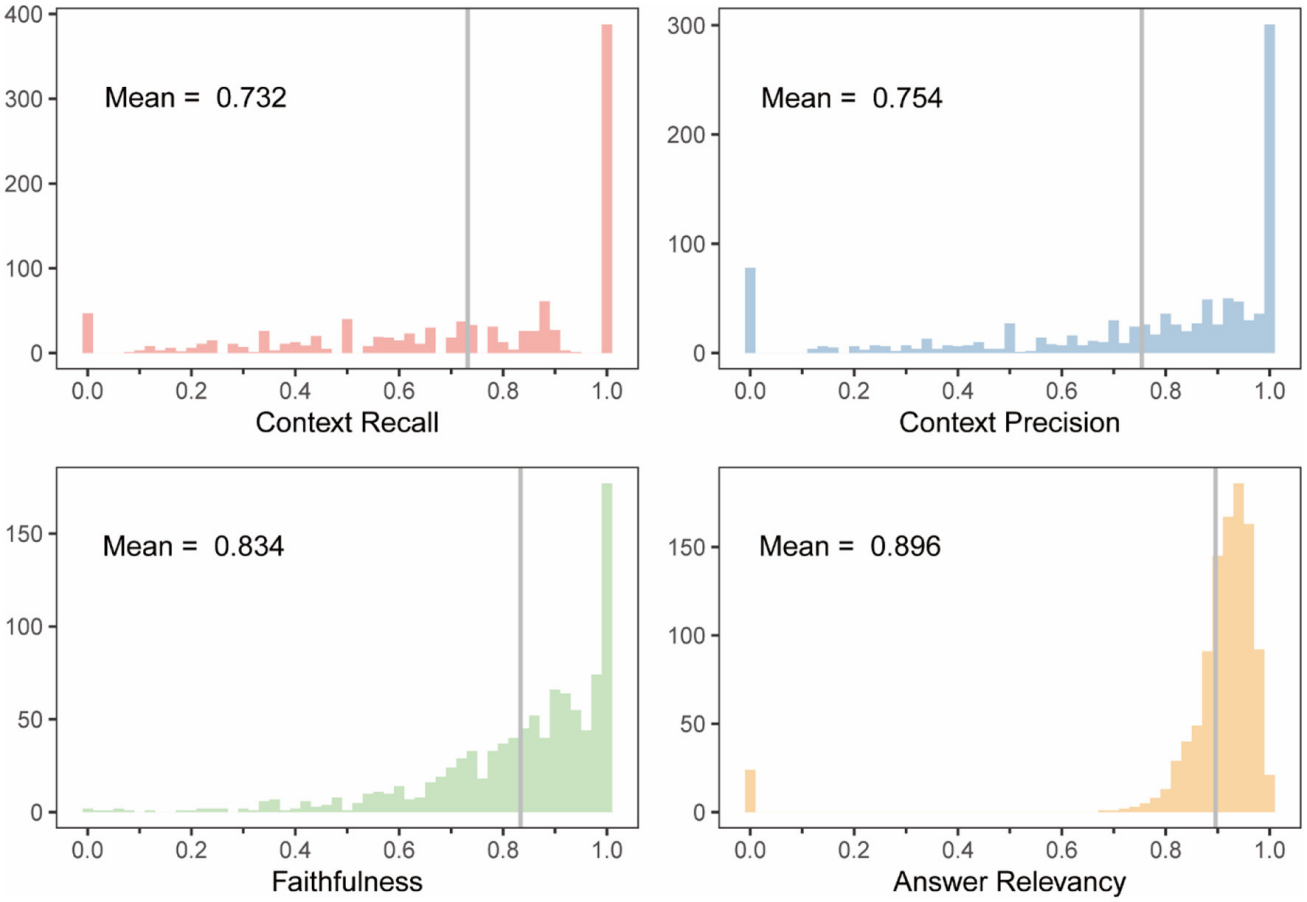
Score distributions of four evaluation metrics for the RAG-based LLM including context recall, context precision, faithfulness, and answer relevancy. The gray vertical lines indicate the mean values of each distribution.

To evaluate model performance, we calculated four metrics: context recall, context precision, faithfulness, and answer relevancy (Supplementary Table S2) based on the retrieved references and generated responses. The first two metrics assessed the precision and relevance of the retrieved references, while the latter two evaluated the relevance and factuality of the generated answers. The model achieved scores of 0.732, 0.754, 0.834, and 0.896 (Figure 6), respectively, demonstrating robust performance in both retrieval and generation tasks. To further illustrate the system's capabilities, we also assessed its performance on complex queries that require synthesizing information from multiple sources within the knowledge base (see Supplementary Figure S4 for examples). For instance, one such query was “Compare the efficacy of ultrasonic treatment and chemical algicides for controlling Microcystis aeruginosa blooms in drinking water reservoirs, considering potential side effects on water quality.” The system demonstrated the ability to retrieve relevant information from different articles and provide a synthesized response, supported by citations.

## 4 Discussion

Chemical algicides are critical emergency measures for HAB control. AlgicideDB provides a comprehensive toolkit for systematic research and management of algicides while establishing a data foundation for HAB control. By focusing on algicidal molecules and providing comprehensive, structured information, AlgicideDB facilitates systematic data analysis, accelerates the discovery and optimization of novel algicides, and supports decision-making in HAB management.

Our analysis of algicidal molecules in the database revealed a predominant focus on toxic cyanobacteria and dinoflagellates due to their toxin production and severe ecological impacts, with a notable emphasis on species relevant to East Asian waters (e.g., *Microcystis aeruginosa, Heterosigma akashiwo, Cochlodinium polykrikoides*). However, research on algicides targeting other algal phyla, such as *Aureococcus anophagefferens* (causing brown tides) and *Sargassum* (causing golden tides), remains relatively scarce. Expanding the scope of investigation to include a broader range of harmful algae is crucial for addressing the complex and dynamic nature of HABs. Future development of the database will focus on taxonomic diversification and geographic expansion through international research collaborations and community contributions to build a more comprehensive global resource for HAB management.

While numerous algicidal molecules demonstrate promising efficacy, these compounds primarily derive from single-species laboratory studies, limiting their extrapolation to complex natural ecosystems. When evaluating algicide applicability, ecological safety should be comprehensively considered. In this study, we identified potential toxicity risks through in silico predictions, with our assessment of potential toxicity to non-target aquatic organisms relying primarily on computational predictions using tools like ADMETlab 3.0. Although such computational toxicology tools offer valuable toxicity predictions, these models may struggle with novel chemical scaffolds or specific modes of action not well-represented in their training data, potentially affecting the confidence of predictions across different compound classes. Future research could utilize *in silico* toxicity prediction for preliminary screening to increase the probability of identifying promising candidates. For those candidate molecules, besides validating their algicidal efficacy, toxicity testing on non-target organisms is equally important, including acute toxicity, chronic toxicity, and sublethal effects. Additionally, in natural aquatic environments, these compounds interact with diverse microbial communities, potentially causing unpredicted ecological effects. Therefore, prior to field implementation, ecological safety assessments in simulated or natural systems are essential to validate laboratory findings and evaluate potential risks.

Moreover, AlgicideDB provides algicidal activity scoring and HAB-related question-answering capabilities. The algicide-likeness scoring function offers an efficient preliminary screening tool for prioritizing potential algicides within large chemical libraries due to its straightforward and rapid calculation. Its ability to rank compounds irrespective of strict adherence to general pesticide-likeness rules is a key advantage, particularly when compared to simply filtering based on descriptor ranges. While the current QEA model shows promise, its performance is inherently limited by the quality and coverage of the data within AlgicideDB. By integrating larger datasets and employing advanced machine learning techniques, future research could enhance model performance and generalizability, thereby accelerating the discovery and optimization of novel algicides. Meanwhile, the integration of RAG-based language models demonstrates potential in HAB-related question-answering and decision-making support. While our initial evaluation using automatically generated question-answer pairs from the knowledge base provided preliminary insights, future research will prioritize user-centered evaluations involving environmental managers and researchers, as their real-world queries will more authentically measure the system's effectiveness. To improve the system's ability to handle complex, multi-faceted queries, subsequent research will explore the integration of a knowledge graph. Representing entities (e.g., specific algal species, algicides, environmental conditions) and their relationships explicitly could enable more accurate and nuanced information retrieval and synthesis. As the field of HAB research evolves, dynamic updates to the knowledge base will be necessary to maintain reliability and enhance real-world applicability.

In conclusion, AlgicideDB provides a foundation for advancing algicide development and HAB management. By systematically curating and analyzing data on algicide sources, chemical structures, target algae, and ecological impacts, this platform facilitates the design of more effective and environmentally sustainable solutions. With the rapid growth of algicide-related literature, future developments could focus on implementing automated data extraction tools powered by large language models with human verification to efficiently capture research advances while maintaining data integrity. Additionally, establishing regular update protocols will continuously enrich the database and enhance its practical utility. These optimizations will position AlgicideDB as a valuable long-term resource for algicide research and HAB management, ultimately contributing to aquatic ecosystem protection and sustainable water resource management.

## Data Availability

The original contributions presented in the study are publicly available. Codes and data for RAG are available at Github (https://github.com/zzzqiii/ADB).
