# Supplementary material for: AlgicideDB: a comprehensive database enhanced by large language models for algicide management and discovery
Source: Front Microbiol. 2025 Jun 18;16:1611403. doi: 10.3389/fmicb.2025.1611403 (PMC12213500; doi:10.3389/fmicb.2025.1611403)
Supplement: Supplementary file 1 [file Supplementary_file_1.docx]

Supporting Information for

**AlgicideDB: A Comprehensive Database Enhanced by Large Language Models for Algicide Management and Discovery**

Zhangqi Zuo^1,2^, Jing Hu^3^, Chaowei Zhang^2^, Zuoqi Wang^2^, Lei Chen^1^, Fei Li^2^, Xi Xiao^1*^

^1^ Ocean College, Zhejiang University, 1 Zheda Road, Zhoushan, Zhejiang 316021, China

^2^ College of Agriculture and Biotechnology, Zhejiang University, Hangzhou, 310058, China

^3^ State Key Laboratory of Marine Pollution, City University of Hong Kong, 999077, Hong Kong

^*^ Corresponding author: Prof. Xi Xiao, Email: [prana@zju.edu.cn](mailto:prana@zju.edu.cn)

Contents of this file

Figure S1 to S4

Table S1 to S7

Reference


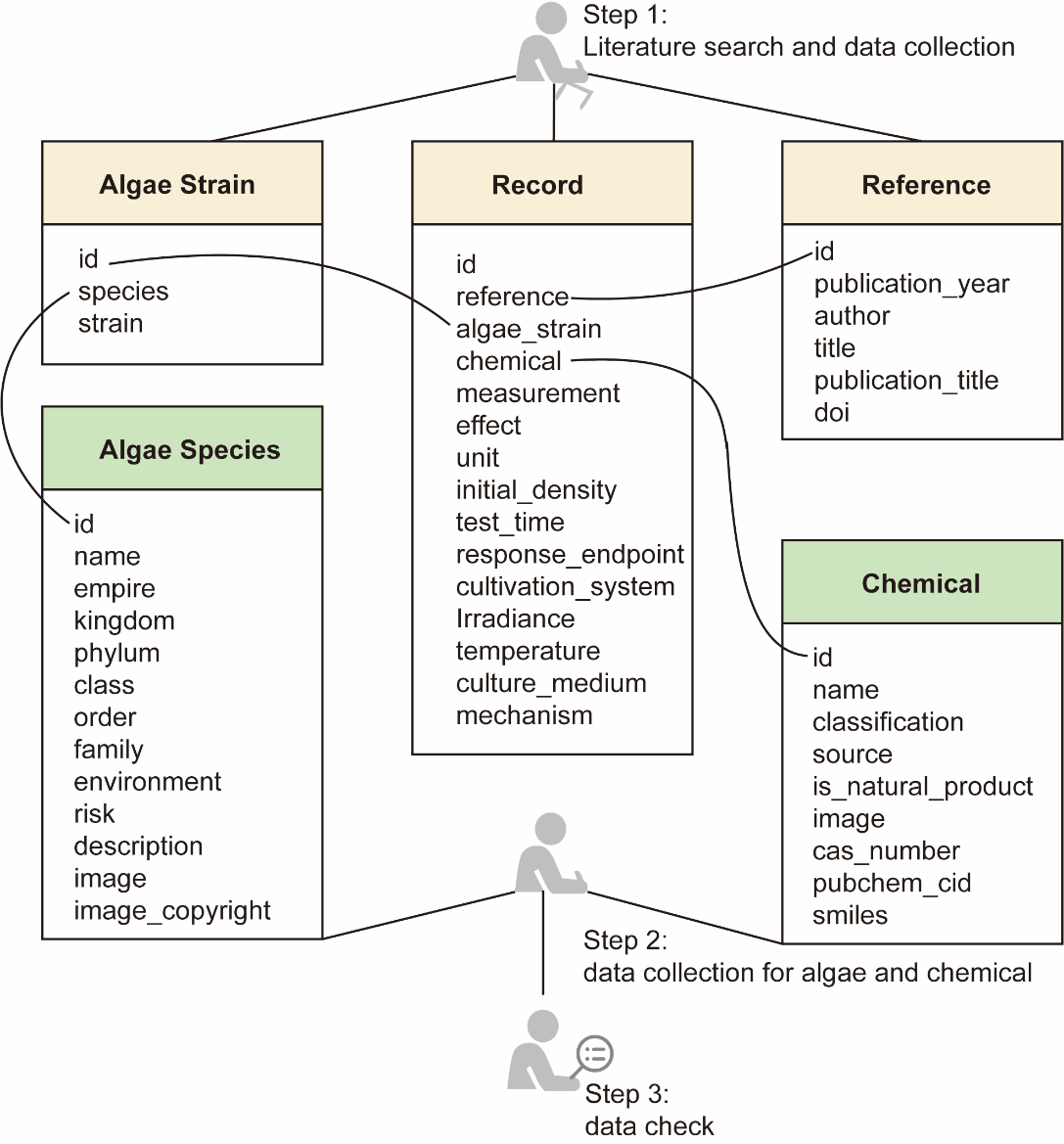


**Fig. S1.** Workflow of data curation.


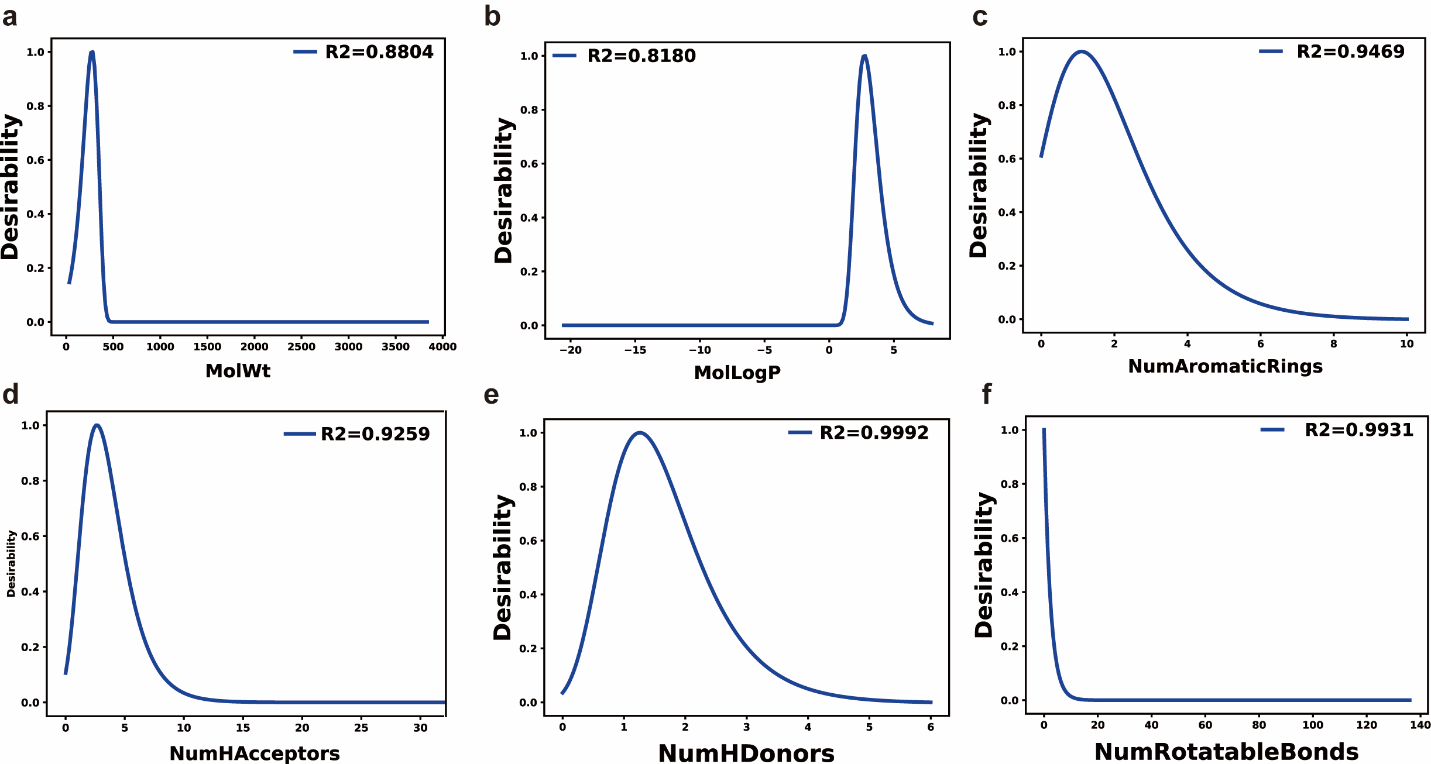


**Fig. S2.** Desirability functions plots of six molecular descriptors.


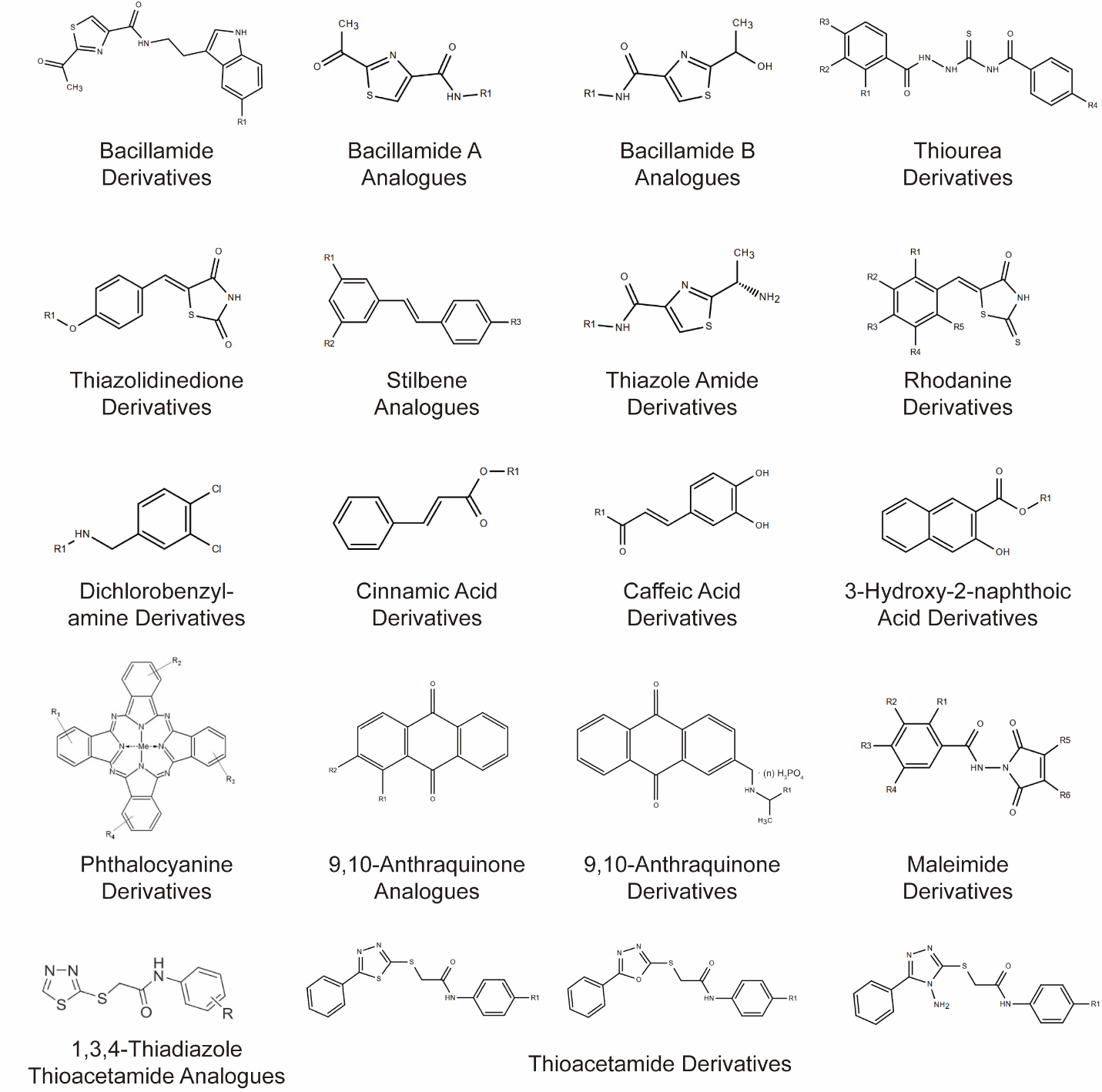


**Fig. S3.** Core Structures of Synthetic Algicidal Compounds


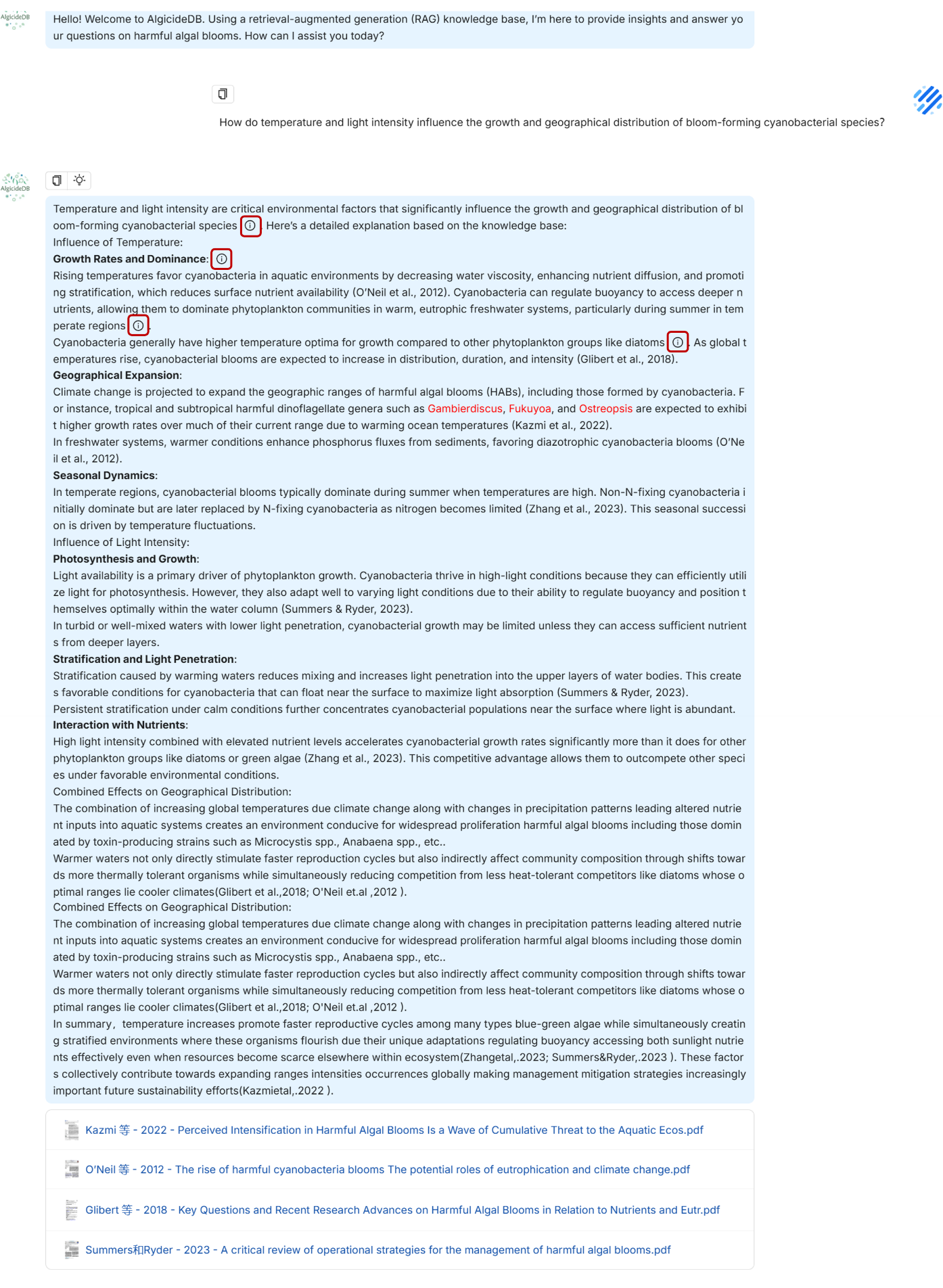


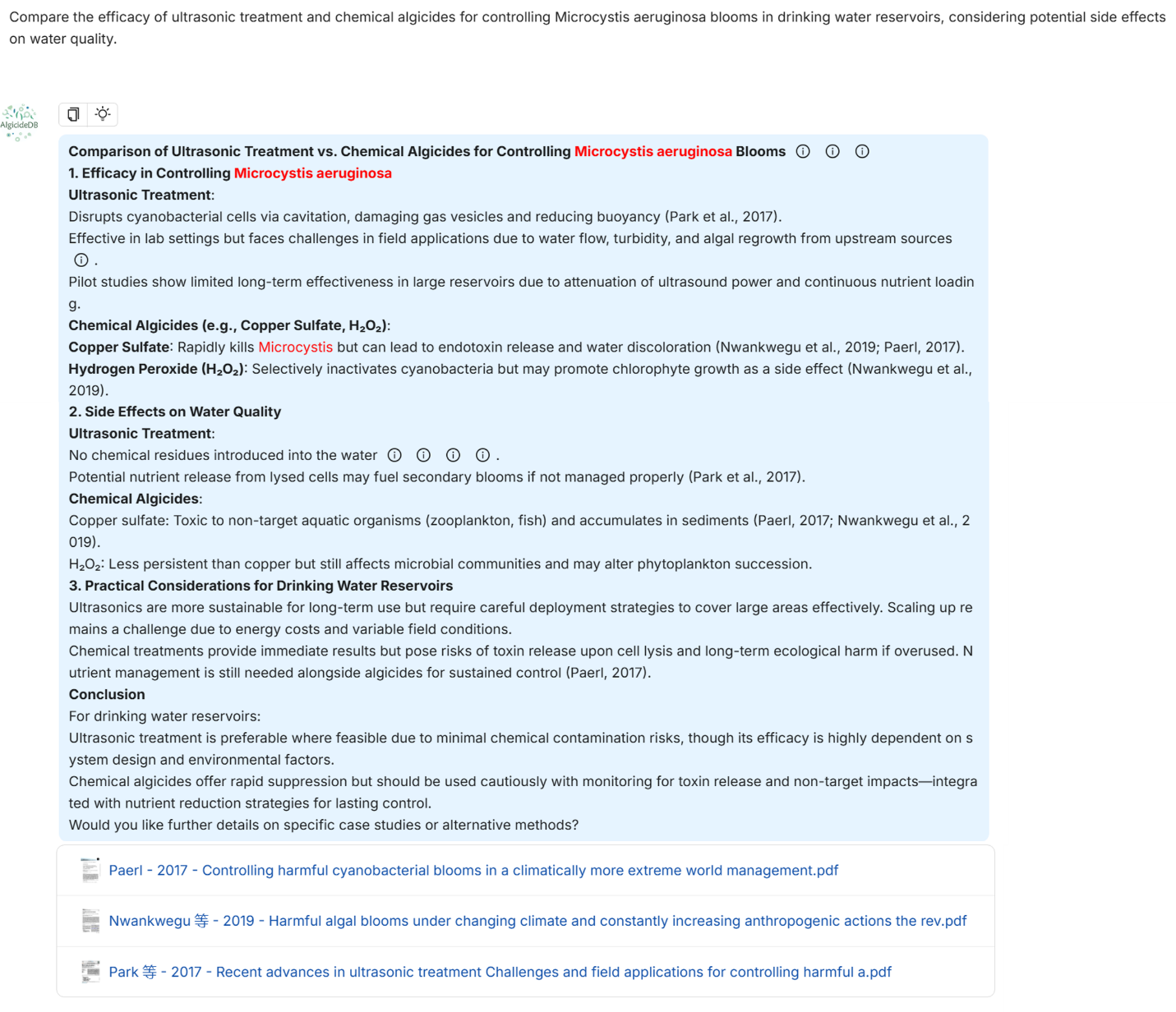


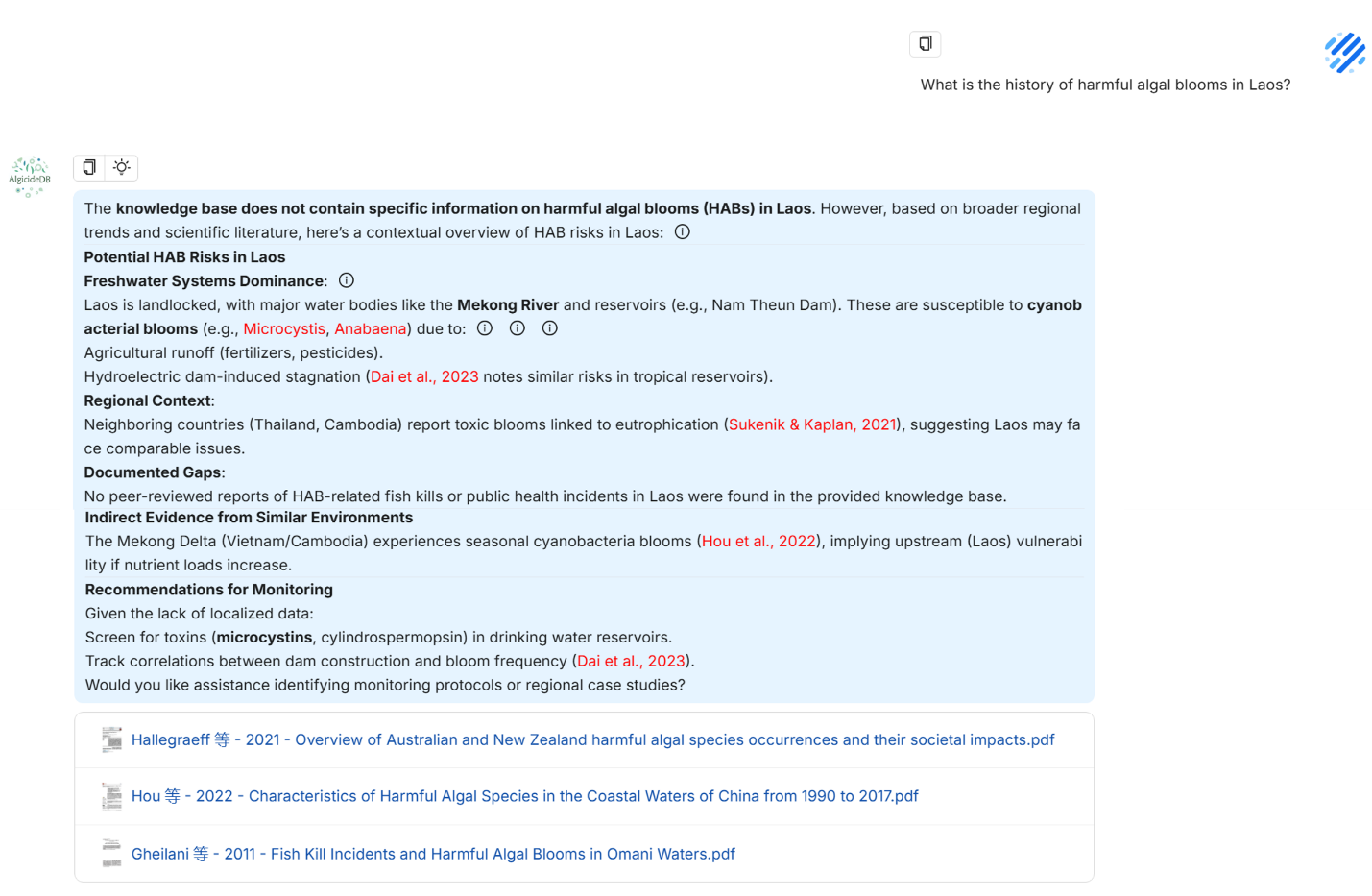


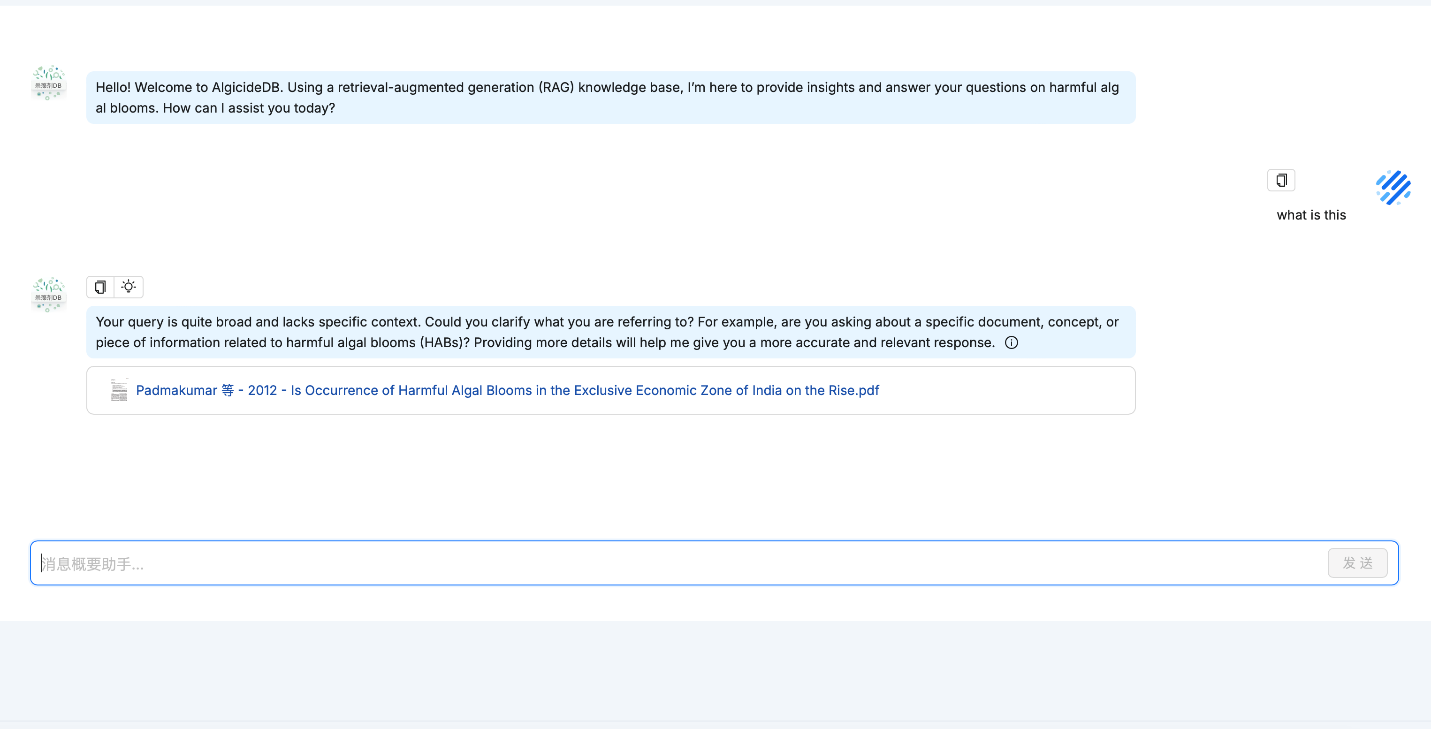


**Fig. S4.** Examples of RAG-based LLM responding to a HABs-related query.

**Table S1.** Coefficients used to fit desirability functions for QEA.

| Property | o | a | b | c |
| --- | --- | --- | --- | --- |
| MolWt | 0.052 | 23.989 | 274.103 | -83.679 |
| MolLogP | 1.010 | 28.173 | 2.723 | 0.876 |
| NumAromaticRings | -2.208 | 90.836 | 1.102 | 1.294 |
| NumHDonors | 6.238 | 119.762 | 1.260 | 0.699 |
| NumHAcceptors | -0.229 | 60.165 | 2.643 | 1.681 |
| NumRotatableBonds | 2.200 | 49.964 | 1.251 | 1.772 |

**Table S2.** Descriptions of evaluation metrics for the RAG-based LLM.

| Metric | Description |
| --- | --- |
| Context Recall | Proportion of relevant chunks successfully retrieved from the database. Higher values indicate more comprehensive retrieval. |
| Context Precision | Average precision@k, where precision@k is the ratio of relevant chunks to the total retrieved chunks up to rank k. Higer values indicate greater relevance of the retrieved contexts. |
| Faithfulness | Factual consistency of the generated answer with the retrieved context, scaled between 0 and 1. Higher values indicate better alignment. |
| Answer Relevancy | Relevance of the generated answer to the given prompt, calculated using cosine similarity between user input and reverse-engineered questions based on the response. Higher scores indicate better relevancy of the responses. |

**Table S3.** Summary of plant-sourced algicide and target algae. Green, yellow, and blue backgrounds indicate sources from aquatic plant, terrestrial plant, and macroalgae, respectively.

| Reference | Source | Algicide | Test algae |
| --- | --- | --- | --- |
| Ref. (Cheng et al., 2017) | *Eichhornia crassipes* | N-Phenyl-1-naphthylamine | *Microcystis sp.* |
| Ref. (Pei et al., 2018) | *Eichhornia crassipes* | N-phenyl-2-naphthylamine | *Scenedesmus quadricauda,*  *Microcystis aeruginosa* |
| Ref. (DellaGreca et al., 2001) | *Potamogeton natans* | 19-Acetoxy-15,16-epoxy-8(17),13(16),14-ent-labdatrien-20-al,  19,20-Dihydroxy-15,16-epoxy-8(17),13(16),14-ent-labdatriene,  10α,19-Dihydroxy-15,16-epoxy-8(17),13(16),14-nor-ent-labdatriene,  12(S)-Hydroxy-15,16-epoxy-8(17),13(16),14-ent-labdatrien-20,19-olide,  15,16-Epoxy-8(17),13(16),14-ent-labdatrien-20,19-olide | *Raphidocelis subcapitata* |
| Ref. (Li and Hu, 2005) | *Phragmites communis* | Ethyl 2-methylacetoacetate | *Microcystis aeruginosa,*  *Chlorella pyrenoidosa* |
| Ref. (Nakai et al., n.d.) | *Phragmites australis* | Gallic acid, Myristic acid,  Caffeic acid, Vanillic acid, Protocatechuic acid,  p-Coumaric acid,  Sinapic acid,  Syringic acid,  Pelargon acid | *Microcystis aeruginosa,*  *Phormidium tenue* |
| Ref. (Gao et al., 2011) | *Hydrilla verticillata* and Vallisneria spiralis | Vanillic acid,  Protocatechuic acid,  Ferulic acid, Caffeic acid | *Microcystis aeruginosa* |
| Ref. (Zhao et al., 2022) | Lotus | Hordenine, Neferine, Nuciferine | *Microcystis aeruginosa* |
| Ref. (Hong et al., 2010) | giant reed (*Arundo donax*) | Gramine | *Microcystis aeruginosa* |
| Ref. (Mary et al., 2024) | giant reed (*Arundo donax*) | Ellipticine | *Prymnesium parvum,*  *Chlorella sorokiniana* |
| Ref. (Zhu et al., 2010) | *Myriophyllum spicatum* | Gallic acid, Pyrogallol,  (+)-Catechin, Ellagic acid | *Raphidocelis subcapitata,*  *Microcystis aeruginosa* |
| Ref. (Nakai et al., 2005) | *Myriophyllum spicatum* | cis-9-Octadecenoic acid,  cis-6-Octadecenoic acid,  Nonanoic acid | *Microcystis aeruginosa* |
| Ref. (Wang et al., 2014) | *Elodea nuttallii* | Dihydroactinidiolide,  Beta-ionone | *Microcystis aeruginosa* |
| Ref. (Kang et al., 2020) | Cattail (*Typha angustifolia*) | 100-day extract of fresh cattail | *Microcystis aeruginosa* |
| Ref. (Effiong et al., 2022) | Canola straw | Cinnamic acid, Linoleic acid,  Succinic acid, Methylmalonic acid, Chlorogenic acid, L-phenylalanine,  3-Indoleacrylic acid, 1,2-Benzenediol | *Prorocentrum donghaiense* |
| Ref. (Hu et al., 2023) | Canola Straw decomposed by *Trametes versicolor* | Sphinganine, Phytosphingosine,  DL-alanyl-DL-phenylalanine,  l-Methionine,  Choline alfoscerate,  3-Hydroxyanthranilic acid,  Sphingosine,  N-acetylsphingosine | *Skeletonema costatum,*  *Chlorella sp.,*  *Isochrysis galbana,*  *Amphidinium carterae,*  *Microcystis aeruginosa,*  *Phaeocystis globosa,*  *Heterosigma akashiwo* |
| Ref. (Xiao et al., 2014) | Barley straw (*Hordeum vulgare*) | Salcolin B, Salcolin A | *Microcystis aeruginosa* |
| Ref. (Murray et al., 2010) | Barley straw | Benzoic acid,  2-Methylbutanoic acid,  3-Methylbutanoic acid,  Hexanoic acid, p-Cresol,  Trans-cinnamic acid, Acetophenone, 2-Phenyl-phenol, Heptanoic acid, Benzyl cyanide, Benzaldehyde | *Chlorella vulgaris,*  *Scenedesmus subspicatus,*  *Microcystis aeruginosa* |
| Ref. (Park et al., 2009) | Rice hull | Beta-sitosterol-beta-D-glucoside,  Dicyclohexanyl orizane | *Microcystis aeruginosa* |
| Ref. (Park et al., 2006) | Rice straw | Rice straw extract | *Microcystis aeruginosa* |
| Ref. (Park et al., 2020) | Walnut hull | Juglone | *Microcystis aeruginosa,*  *Stephanodiscus hantzschii* |
|  |  |  |  |
| Ref. (Zhang et al., 2013) | *Salvia miltiorrhiza* | Neo-przewaquinone A | *Microcystis aeruginosa,*  *Chlorella pyrenoidosa,*  *Scenedesmus obliquus* |
| Ref. (Shao et al., 2013) | *Sanguinaria canadensis* | Sanguinarine | *Microcystis aeruginosa* |
| Ref. (Xu et al., 2022) | *Lancea tibetica* (Mazaceae) | 5, 4'-dihydroxyflavone | *Microcystis aeruginosa,*  *Phaeocystis globosa* |
| Ref. (Purcaro et al., 2009) | *Swinglea glutinosa* | 5-Methoxyseselin,  6-(3,3-Dimethylallil) seselin, Citibrasine, Seselin | *Raphidocelis subcapitata,*  *Oscillatoria perornata* |
| Ref. (Huang et al., 2024b) | *Cinnamomum camphora* | Camphor, Borneol,  Eucalyptol, Linalool | *Microcystis aeruginosa* |
| Ref. (Kim, Young Do et al., 2013) | *Cinnamomum loureiroi* | coumarin, cinnamaldehyde, hydroxylcoumarin, coumaric acid | *Navicula annexa,*  *Ulva pertusa* |
| Ref. (Liang et al., 2022) | *Lpomoea cairica* | Lpomoea cairica extracts | *Phaeocystis globosa* |
| Ref. (Sang et al., 2024) | *Artemisia annua* | Artemisinin | *Microcystis aeruginosa* |
| Ref. (Wong et al., 2024) | *Kandelia obovata* | Kandelia obovata leaf extract | *Karenia mikimotoi,*  *Alexandrium tamarense* |
| Ref. (Meepagala et al., 2005) | *Ruta graveolens* | rutacridone epoxide,  gravacridondiol | *Raphidocelis subcapitata,*  *Oscillatoria perornata* |
| Ref. (Kim et al., 2006) | *Polygonatum odoratum* var. pluriflorum Owhi | l-2-azetidinecarboxylic acid, Azetidine | *Cochlodinium polykrikoides,*  *Microcystis aeruginosa,*  *Anabaena affinis* |
| Ref. (Zhou et al., 2007) | *Rhizoma coptidis* Huang Lian and *Semen arecae* Bing Lang | Rhizoma coptidis Huang Lian extracts,  areca seed extracts | *Alexandrium tamarense* |
| Ref. (Zhou et al., 2008) | Garlic (*Allium sativum*) | Diallyl trisulfide | *Alexandrium tamarense* |
| Ref. (Yi et al., 2012) | *Salvia miltiorrhiza*, *Acorus tatarinowii*, *Polygonum cuspidatum*, *Crataegus pinnatifida*, *Phellodendron amurense* | Extracts of the rhizome of *Acorus tatarinowii*, the fruits of *Crataegus pinnatifida*, the rhizome of *Polygonum cuspidatum*, the root of *Salvia miltiorrhiza*, the cortex of *Phellodendron amurense* | *Microcystis aeruginosa* |
| Ref. (El Bouaidi et al., 2020) | *Vicia faba* and *Opuntia ficus indica* | Bean extract, Cactus extract | *Microcystis aeruginosa* |
| Ref. (Tebaa et al., 2021) | *Thymus maroccanus*, *Origanum compactum*, *Artemisia herba-alba* | Extracts of the fresh leaves of *Thymus maroccanus*, *Origanum compactum*, and *Artemisia herba-alba* | *Microcystis aeruginosa* |
| Ref. (Kong et al., 2006) | *Lantana camara* | Lantadene A,  Lantadene B | *Microcystis aeruginosa* |
| Ref. (Hao et al., 2022) | Semiarundinaria densiflora (Rendle) T.H. Wen | Bamboo extract | *Microcystis aeruginosa* |
| Ref. (Bazes et al., 2009) | Sargassum muticum (Yendo) Fensholt | Palmitic acid | *Ulva lactuca,*  *Cylindrotheca closterium* |
| Ref. (Cho, 2013) | Sargassum horneri | Chromanol S2, S1, S3, S4, S5, S6 | *Navicula annexa,*  *Ulva pertusa* |
| Ref. (Alamsjah et al., 2005) | Ulva fasciata | Octadeca-6,9,12,15-tetraenoic acid, Hexadeca-4,7,10,13-tetraenoic acid, Linolenic acid | *Heterosigma akashiwo* |
| Ref. (Alamsjah et al., 2008) | *Ulva fasciata* and *U. pertusa* | Arachidic acid, Erucic acid,  Oleic acid, 11-Eicosenoic acid, Behenic acid, Palmitelaidic acid, Palmitoleic acid, Stearic acid,  α-Linolenic acid, Linoleic acid, Octadecatetraenoic acid, Docosahexaenoic acid, Eicosapentaenoic acid,  Palmitic acid, Arachidonic acid, Hexadecatetraenoic acid | *Heterosigma akashiwo,*  *Chattonella marina* |
| Ref. (Hirao et al., 2012) | Brown Alga *Ishige sinicola* | 1-O-palmitoyl-3-O-(6’-sulfo-D-quinovopyranosyl)-sn-glycerol,  Oleic acid, Arachidonic acid, Octadeca-6,9,12,15-tetraenoic acid, Linolenic acid | *Karenia mikimotoi,*  *Alexandrium catenella,*  *Heterosigma akashiwo,*  *Chattonella marina* |
| Ref. (Sun et al., 2017) | *Gracilaria lemaneiformis* | 15-Hydroxymethyl-2, 6, 10,18, 22, 26, 30heptamethyl-14-methylene-17-hentriacontene,  Glycerol monopalmitate,  Gossonorol,  Margaric acid,  p-Hydroxyphenylethanol,  7,10-Epoxy-ar-bisabol-11-ol,  Stigmasterol | *Skeletonema costatum,*  *Prorocentrum donghaiense,*  *Phaeocystis globosa,*  *Heterosigma akashiwo,*  *Karenia mikimotoi* |
| Ref. (Lu et al., 2011) | *Gracilaria lemaneiformis* | 8-Hydroxy-4E,6E-octadien-3-one,  3β-Hydroxy-5α,  6α-Epoxy-7-megastigmen-9-one | *Skeletonema costatum* |
| Ref. (Sun et al., 2021) | *Gracilaria lemaneiformis* | Linoleic acid,  3-Hydroxymethylpyrrolopiperazine-2,5-dione,  Benzene-1,2-propanoic acid,  1-β-D-ribofuranosyluracil,  1-O-Palmitoyl-2-O-palmitoleoyl-3-O-β-Dgalactopyranosyl glycerol | *Skeletonema costatum,*  *Amphidinium carterae,*  *Prorocentrum donghaiense,*  *Phaeocystis globosa,*  *Heterosigma akashiwo, Karenia mikimotoi* |

**Table S4.** Summary of microorganism-sourced algicide and target algae.

| Reference | Source | Algicide | | | | | | Test Algae | |
| --- | --- | --- | --- | --- | --- | --- | --- | --- | --- |
| Ref. (Li et al., 2016) | *Acinetobacter* sp. CMDB-2 | cell-free filtrate | | | *Microcystis aeruginosa* | | | | |
| Ref. (Weiss et al., 2019) | *Aeromonas veronii* | Lumichrome | | | *Microcystis aeruginosa* | | | | |
| Ref. (Liu et al., 2013) | *Aeromonas* sp. FM | L-lysine, Clavulanate | | | *Microcystis aeruginosa* | | | | |
| Ref. (Guo et al., 2016) | *Aeromonas* sp. GLY-2107 | 3-Benzyl-piperazine-2,5-dione,  3-Methylindole | | | *Microcystis aeruginosa* | | | | |
| Ref. (Umetsu et al., 2019) | *Alteromonas* sp. D | Questiomycin C, Questiomycin A, Questiomycin E, Questiomycin D | | | *Karenia mikimotoi,*  *Chattonella marina,*  *Bangia fuscopurpurea,*  *Chaetoceros didymus* | | | | |
| Ref. (Cho, 2012a) | *Alteromonas* sp. KNS-16 | 2-Undecyl-4-uinolone,  2-Undecen-1'-yl-4-quinolone,  6-Heptyl-3-hexyl-4-hydroxyl-2H-pyran-2-one,  3-Hexyl-6-pentyl-4-hydroxyl-2H-pyran-2-one | | | *Cochlodinium polykrikoides,*  *Alexandrium tamarense,*  *Heterosigma akashiwo* | | | | |
| Ref. (Mohamed et al., 2022) | *Bacillus* *flexus* SSZ01 | Bacterial suspension | | | *Raphidiopsis raciborskii* | | | | |
| Ref. (Bedoshvili et al., 2021) | *Bacillus* *mycoides* BS2-15 | Bacterial culture | | | *Ulnaria acus* | | | | |
| Ref. (Quan et al., 2021)  Ref. (Zhuang et al., 2018), Ref. (Zhao et al., 2014) | *Bacillus* sp. B1 | N-acetylhistamine, L-histidine,  Urocanic acid,  Ortho-tyrosine  Hypoxanthine | | | *Phaeocystis globosa,*  *Prorocentrum donghaiense,*  *Skeletonema costatum,*  *Heterosigma akashiwo* | | | | |
| Ref. (Jeong et al., 2003) | *Bacillus* sp. SY-1 | Bacillamide | | *Cochlodinium polykrikoides* | | | | | |
| Ref. (Jeong and Son, 2021) | *Bacillus* sp. SY-1 | mycosubtilins 1084, mycosubtilins 1056, mycosubtilins 1070 | | *Prorocentrum minimum,*  *Heterosigma akashiwo,*  *Prorocentrum dentatum,*  *Prorocentrum micans,*  *Alexandrium tamarense,*  *Akashiwo sanguinea,*  *Prorocentrum triestinum,*  *Gymnodinium impudicum,*  *Heterocapsa triquetra,*  *Cochlodinium polykrikoides,*  *Chattonella marina* | | | | | |
| Ref. (Li et al., 2015) | *Bacillus* sp. Lzh-5 | 3-Isopropyl-hexahydropyrrolo[1,2-a] pyrazine-1,4-dione | | | | *Microcystis aeruginosa* | | | |
| Ref. (Wu et al., 2017) | *Bacillus* sp. S51107 | cyclo(Pro-Phe), indole-3-carboxaldehyde | | | *Microcystis aeruginosa* | | | | |
| Ref. (Yang et al., 2022) | *Bacillus subtilis* | Bacillomycin D | | *Skeletonema costatum* | | | | | |
| Ref. (Kim et al., 2015) | *Brachybacterium* sp. YS-3 | 1-Acetyl-β-carboline | | *Fibrocapsa japonica,*  *Prorocentrum minimum,*  *Heterosigma akashiwo,*  *Alexandrium catenella,*  *Prorocentrum micans,*  *Akashiwo sanguinea,*  *Gymnodinium impudicum,*  *Cochlodinium polykrikoides,*  *Chattonella marina,*  *Scrippsiella trochoidea* | | | | | |
| Ref. (Liu et al., 2024) | *Brevibacillus* sp. | 4-Acetamidobutyric acid,  8-Hydroxyquinoline | | *Microcystis aeruginosa* | | | | | |
| Ref. (Sun et al., 2004) | *Candida bombicola* ATCC22214 | Sophorolipid | | | *Heterosigma akashiwo,*  *Cochlodinium polykrikoides,*  *Alexandrium tamarense* | | | | |
| Ref. (Guo et al., 2015) | *Chryseobacterium* sp. GLY1106 | cyclo(Pro-Leu), cyclo(4-OH-Pro-Leu) | | | *Microcystis aeruginosa* | | | | |
| Ref. (Zhang et al., 2022) | *Enterobacter hormaechei* F2 | Prodigiosin | | *Microcystis aeruginosa* | | | | | |
| Ref. (Lu et al., 2021) | *Enterobacter* sp. EA-1 | Enterobacter sp. EA-1 cultures | | | *Oscillatoria tenuis,*  *Oscillatoria sp.,*  *Planktothrix agardhii,*  *Microcystis aeruginosa,*  *Chlorella vulgaris,*  *Microcystis wesenbergii* | | | | |
| Ref. (Hagmann and Jüttner, 1996) | *Fischerella muscicola* | Fischerellin A | | *Scenedesmus subspicatus,*  *Scenedesmus obliquus,*  *Synechococcus sp.* | | | | | |
| Ref. (Zhang et al., 2017) | *Hahella* sp. KA22 | Prodigiosin | | | *Phaeocystis globosa* | | | | |
| Ref. (Zhang et al., 2020b) | *Halobacillus* sp. P1 | Culture supernatant | | | *Skeletonema costatum* | | | | |
| Ref. (Abd ElTawab et al., 2021) | *Lactobacillius* *sakei* | Culture supernatant | | | *Trichormus variabilis* | | | | |
| Ref. (Cho, 2012c)  Ref. (Cho, 2012b) | *Leucothrix mucor* | 13-Acetate-17-(1,5-dimethylhexane)-cholest-7-en-3,5,6,15-tetraol,  17-(1,2-Dihydroxyl-5-methyl-hexane)-2,3-dihydroxyl-cholest-4-en-6-one,  Giffinisterone B, Oleamide | | *Ulva pertusa,*  *Navicula annexa* | | | | | |
| Ref. (Zhang et al., 2023) | *Microbulbifer* sp. RZ01 | 3,3’,5,5’-Tetrabromo-2,2’-biphenyldiol | *Phaeodactylum tricornutum* | | | | | |  |
| Ref. (Ozaki et al., 2008) | *Microcystis* VOC | Menthone,  Carvacrol,  Perillaldehyde,  2-Methylisoborneol, cinnamaldehyde,  β-Cyclocitral,  Geraniol, Linalool,  Geosmin, Beta-ionone,  3-Methyl-1-butanol,  Vanillin, Menthol,  Thymol | | | *Microcystis sp.* | | | | |
| Ref. (Blom et al., 2006) | *Nostoc* 78-12A | Nostocarboline | | *Kirchneriella contorta,*  *Synechococcus sp.,*  *Microcystis aeruginosa* | | | | | |
| Ref. (Bagchi, 1995) | *Oscillatoria late-virens* | C_29_H_48_O_3_ | | *Synechococcus sp.* | | | | | |
| Ref. (Wang et al., 2021) | *Paenibacillus* sp. SJ-73 | Fermentation filtrate | | | *Anabaena sp.,*  *Aphanizomenon flos-aquae,*  *Microcystis aeruginosa* | | | | |
| Ref. (Ding et al., 2021) | *Paracoccus homiensis* | Cell-free filtrate | | | *Karenia mikimotoi* | | | | |
| Ref. (Le et al., 2022) | *Paucibacter aquatile* DH15 | Cell cultures | | *Microcystis aeruginosa* | | | | | |
| Ref. (Wu et al., 2011) | Periphyton biofilms | 3-Oxo-α-ionone, indole | | | *Microcystis aeruginosa* | | | | |
| Ref. (Wang and Seyedsayamdost, 2017) | *Phaeobacter inhibens* | Roseochelin B | | *Emiliania huxleyi* | | | | | |
| Ref. (Whalen et al., 2018) | *Pseudoalteromonas* *piscicida* (A757) | 2,3,4,5-Tetrabromo-1H-pyrrole | | | *Mantoniella squamata,*  *Heterosigma akashiwo,*  *Rhodomonas sp.,*  *Isochrysis galbana,*  *Thalassiosira pseudonana,*  *Thalassiosira oceanica* | | | | |
| Ref. (Harvey et al., 2016) | *Pseudoalteromonas* *piscicida* | 2-Heptyl-4-quinolone | | | *Emiliania huxleyi* | | | | |
| Ref. (Gustafsson et al., 2009)  Ref. (Wang et al., 2005) | *Pseudomonas aeruginosa* | Rhamnolipid biosurfactant | | | *Alexandrium minutum,*  *Karenia brevis,*  *Gonyostomum semen,*  *Pseudo-nitzschia sp.,*  *Heterosigma akashiwo,*  *Prorocentrum dentatum* | | | | |
| Ref. (Zhou et al., 2021) | *Pseudomonas aeruginosa* | 1-Hydroxyphenazine | | | *Microcystis aeruginosa* | | | | |
| Ref. (Rose et al., 2021) | *Pseudomonas protegens* | 2,4-diacetylphloroglucinol,  Pyrrolnitrin,  Orfamide A, Pyoluteorin,  Rhizoxin S2 | | | | | *Chlamydomonas reinhardtii* | | |
| Ref. (Kodani et al., 2002) | *Pseudomonas* sp. K44-1 | Harmane | | *Microcystis viridis,*  *Anacystis marina,*  *Microcystis aeruginosa,*  *Trichormus variabilis,*  *Phormidium jenkelianum,*  *Anabaena cylindrica* | | | | | |
| Ref. (Qi et al., 2021) | *Pseudomonas* sp. QJX-1 | 2,4-Di-tert-butylphenol | | | *Microcystis aeruginosa* | | | | |
| Ref. (Ko et al., 2022) | *Pseudoruegeria* sp. M32A2M | Cyclo[Ala-Gly] | | *Alexandrium catenella* | | | | | |
| Ref. (Cho et al., 2021) | *Pseudoruegeria* sp. M32A2M | Cell cultures | | | *Alexandrium catenella* | | | | |
| Ref. (Li et al., 2021) | *Raoultella* sp. S1 | Culture supernatant | | | *Microcystis aeruginosa* | | | | |
| Ref. (Wang et al., 2013) | *Rhodococcus* sp. p52 | L-Pyroglutamic acid,  DL-Pipecolic acid,  3-Indoleacrylic acid | | | *Microcystis aeruginosa* | | | | |
| Ref. (Fei et al., 2013) | *Serratia* *marcescens* LTH‐2 | prodigiosin | | | *Microcystis aeruginosa* | | | | |
| Ref. (Bhattarai et al., 2007) | *Shewanella oneidensis* SCH0402 | Oleic acid,  2-Hydroxymyristic acid | | | *Ulva pertusa* | | | | |
| Ref. (Hare et al., 2005)  Ref. (Pokrzywinski et al., 2012) | *Shewanella* sp. IRI-160 | Shewanella IRI-160 cultures | | *Pfiesteria piscicida,*  *Gyrodinium instriatum,*  *Prorocentrum minimum,*  *Oxyrrhis marina,*  *Karenia brevis,*  *Alexandrium tamarense,*  *Heterocapsa triquetra,*  *Cochlodinium polykrikoides,*  *Karlodinium veneficum* | | | | | |
| Ref. (Li et al., 2014b) | *Shewanella* sp. Lzh-2 | Hexahydropyrrolo[1,2-a]pyrazine-1,4-dione, 2, 3-indolinedione | | | | *Synechococcus sp.,*  *Microcystis aeruginosa* | | | |
| Ref. (Lin et al., 2016) | *Stenotrophomonas* F6 | Hydroquinone,  Cyclo-(Gly-Pro) | | *Synechococcus sp.,*  *Microcystis aeruginosa* | | | | | |
| Ref. (Liu et al., 2014) | *Stenotrophomonas* *maltophilia* 15 | Siderophore | | | *Anabaena sp.,*  *Microcystis aeruginosa* | | | | |
| Ref. (Cai et al., 2016) | *Streptomyces alboflavus* | Cell extracts | | *Phaeocystis globosa* | | | | | |
| Ref. (Cho and Kim, 2012) | *Streptomyces cinnabarinus* PK209 | Lobocompactol | | | *Ulva pertusa,*  *Navicula annexa* | | | | |
| Ref. (Zhang et al., 2016) | *Streptomyces eurocidicus* JXJ-0089 | Tryptoline,  Tryptamine | | | *Nostoc sp.,*  *Synechococcus sp.,*  *Anabaena sp.,*  *Aphanizomenon sp.,*  *Microcystis sp.* | | | | |
| Ref. (Zeng et al., 2021) | *Streptomyces* *globisporus* G9 | Cell cultures | | | *Synechocystis sp.,*  *Anabaena flos-aquae,*  *Phormidium sp.,*  *Microcystis aeruginosa* | | | | |
| Ref. (Zheng et al., 2013) | *Streptomyces malaysiensis* O4-6 | NIG355 | | *Chlorella vulgaris,*  *Alexandrium tamarense,*  *Phaeocystis globosa* | | | | | |
| Ref. (Kong et al., 2020) | *Streptomyces* sp. HJC-D1 | Fermented cultures | | | *Microcystis aeruginosa* | | | | |
| Ref. (Luo et al., 2013) | *Streptomyces* sp. L74 | 2-Hydroxy-12-oleanene-3,28-O-D-glucopyranosyl | | | *Microcystis aeruginosa* | | | | |
| Ref. (Yu et al., 2018) | *Streptomyces* sp. U3 | Fermentation broth | | *Prorocentrum donghaiense,*  *Platymonas helgolandica,*  *Skeletonema costatum,*  *Cylindrotheca closterium,*  *Heterosigma akashiwo,*  *Phaeocystis globosa,*  *Microcystis aeruginosa,*  *Alexandrium minutum,*  *Dunaliella salina,*  *Chlorella vulgaris,*  *Thalassiosira pseudonana,*  *Amphiprora alata,*  *Nannochloropsis sp.,*  *Thalassiosira weissflogii,*  *Platymonas subcordiformis,*  *Scrippsiella trochoidea* | | | | | |
| Ref. (Hong and Cho, 2013) | *Streptomyces violaceoruber* SCH-09 | 3-Octa-1'-enyl4-methylfuran-2(5H)-one, 3-octa-1′,3′-dienyl-4-methylfuran-2(5H)-one | | | *Ulva pertusa,*  *Navicula annexa* | | | | |
| Ref. (Zhang et al., 2020a) | *Sulfitobacter* *porphyrae* ZFX1 | Cell-free supernatants | | | *Prorocentrum donghaiense* | | | | |
| Ref. (Hu et al., 2021) | *Sulfitobacter* *pseudonitzschiae* H46 | Cell cultures | | | *Chattonella marina,*  *Alexandrium tamarense* | | | | |
| Ref. (Lu et al., 2016) | *Thalassospira* sp. ZR-2 | Benzoic acid | | | *Karenia mikimotoi* | | | | |
| Ref. (Ouyang et al., 2021) | *Vibrio* *brasiliensis* H115 | Fermentation Products | | | *Akashiwo sanguinea* | | | | |
| Ref. (Li et al., 2014a) | *Vibrio* sp. BS02 | Palmitoleic acid | | *Alexandrium tamarense* | | | | | |
| Ref. (Yoshikawa et al., 2000) | *Vibrio* sp. C-979 | β-Cyano-L-alanine | | | *Microcystis viridis,*  *Synechococcus sp.,*  *Microcystis aeruginosa,*  *Oscillatoria amphibia,*  *Entophysalis deusta* | | | | |
| Ref. (Wang et al., 2012) | *Vibrio* sp. DHQ25 | Purified protein P7 | | | *Alexandrium tamarense* | | | | |
| Ref. (Wang et al., 2020) | *Vibrio* sp. | Spray-dried broth | | | *Akashiwo sanguinea* | | | | |
| Ref. (Nakashima et al., 2006) | strain MS-02–063 | PG-L-1 | | *Heterosigma akashiwo,*  *Heterocapsa circularisquama,*  *Alexandrium tamarense,*  *Gymnodinium impudicum,*  *Cochlodinium polykrikoides* | | | | | |
| Ref. (Benegas et al., 2021) | strain 383 from Antarctica | Cell-free extracts | | *Microcystis aeruginosa* | | | | | |

**Table S5.** Summary of animal-sourced algicides and test algae.

| Reference | Source | Algicide | Test Algae |
| --- | --- | --- | --- |
| Ref. (Feng et al., 2013) | sponge *Axinella* sp. | Debromohymenialdisine, Hymenialdisine | *Ulva prolifera* |

**Table S6.** Summary of synthetic algicides and test algae.

| Reference | Algicide | Test Algae |
| --- | --- | --- |
| Ref. (Churro et al., 2009) | Bacillamide Derivatives | *Microcystis aeruginosa*  *Aphanizomenon gracile*  *Anabaena circinalis*  *Anabaena* sp.  *Anabaenopsis circularis*  *Nodularia spumigena*  *Leptolyngbya* sp.  *Planktothrixrubescens*  *Ankistrodesmus falcatus*  *Scnedesmus obliquus*  *Chlamydomonas* sp.  *Tetraselmis suecica*  *Phaeodactylum tricornutum*  *Cyclotella* sp.  *Diacronema* sp.  *Nannochloropsis* sp. |
| Ref. (Wang et al., 2017) | Bacillamide A Analogues  Bacillamide B Analogues | *Skeletonema costatum*  *Gymnodinium catenatum*  *Mycrocyctis aeruginosa*  *Scenedesmus obliquus*  *Chlorella pyrenoidosa* |
| Ref. (Xiao et al., 2019) | Thiourea Derivatives | *Synechocystis* sp. |
| Ref. (Kim et al., 2010) | Thiazolidinedione Derivatives | *Chattonella marina*  *Heterosigma akashiwo*  *Cochlodinium polykrikoides*  *Amphidinium*sp.  *Phaeodactylum EPV*  *Navicula pelliculosa* |
| Ref. (Mizuno et al., 2008) | Stilbene Analogues | *Oscillatoria perornata*  *Selenastrum capricornutum* |
| Ref. (Wang et al., 2018) | Thiazole Amide Derivatives | *Microcystis aeruginosa*  *Chlorella pyrenoidosa*  *Synechococcus obliqnus* |
| Ref. (Jo et al., 2017) | Rhodanine Derivatives | *Microcystis aeruginosa*  *Aphanizomenon flos-aquae*  *Selenastrum capricornutum* |
| Ref. (Choi et al., 2016) | Dichlorobenzylamine Derivatives | *Heterosigma akashiwo*  *Chattonella marina*  *Heterocapsa circularisquama* |
|  |  | *Cochlodinium polykrikoides* |
| Ref. (Luo et al., 2021) | Cinnamic Acid Derivatives Caffeic Acid Derivatives 3-Hydroxy-2-naphthoic Acid Derivatives | *Microcystis aeruginosa* |
| Ref. (Jančula et al., 2008) | Phthalocyanine Derivatives | *Raphidocelis subcapitata* |
|  |  | *Synechococcus nidulans* |
| Ref. (Nanayakkara and Schrader, 2008) | 9,10-Anthraquinone Analogues | *Oscillatoria perornata* |
|  |  | *Selenastrum capricornutum* |
| Ref. (Schrader et al., 2003) | 9,10-Anthraquinone Derivatives | *Oscillatoria perornata* |
|  |  | *Selenastrum capricornutum* |
| Ref. (Han et al., 2016) | Maleimide Derivatives | *Synechocystis* sp. |
| Ref. (Huang et al., 2023) | 1,3,4-Thiadiazole Thioacetamide Analogues | *Microcystis aeruginosa* |
|  |  | *Synechocystis* sp. |
|  |  | *Chlorella vulgaris* |
| Ref. (Huang et al., 2024a) | Thioacetamide Derivatives | *Microcystis aeruginosa* |
|  |  | *Synechocystis* sp. |
|  |  | *Aphanizomenon* sp. |
|  |  | *Anabaena* sp. |
|  |  | *Nostoc* sp. |
|  |  | *Chlorella vulgaris* |

**Table S7.** Summary of commercial algicides and test algae.

| Reference | Algicide | Ingredient | Test Algae |
| --- | --- | --- | --- |
| Ref. (Hu et al., 2022) | Algimycin® PWF | Copper citrate and copper gluconate | *Karenia brevis* |
| Ref. (Hu et al., 2022)  Ref. (Bishop et al., 2018) | Captain® XTR | Copper ethanolamine complex | *Karenia brevis,*  *Microseira wollei* |
| Ref. (Hu et al., 2022) | SeClear® | Copper sulfate pentahydrate | *Karenia brevis* |
| Ref. (Hu et al., 2022) | GreenClean® Liquid 5.0 | Hydrogen peroxide and peroxyacetic acid | *Karenia brevis* |
| Ref. (Hu et al., 2022) | PAK® 27 | Sodium carbonate peroxyhydrate | *Karenia brevis* |
| Ref. (Hu et al., 2022) | Oximycin® P5 | hydrogen peroxide and peroxyacetic acid | *Karenia brevis* |
| Ref. (Schrader, 2005) | AlgaeFix® | 4.5% of the active ingredient poly[oxyethylene-(dimethyliminio) ethylene(dimethyliminio)ethylene dichloride] | *Oscillatoria perornata,*  *Planktothrix agardhii,*  *Pseudanabaena sp.,*  *Raphidocelis subcapitata* |
| Ref. (Schrader, 2005) | ZeroTol | 27% of the active ingredient hydrogen dioxide | *Oscillatoria perornata,*  *Planktothrix agardhii,*  *Pseudanabaena* sp*.,*  *Raphidocelis subcapitata* |
| Ref. (Schrader et al., 2010) | AquaFrin | Lauryl methyl pyrifrin | *Oscillatoria perornata,*  *Raphidocelis subcapitata* |

**REFERENCES**

Abd ElTawab, M. I., Abdel Gawad, F., Shaban, A. M., Bassem, S., Guerriero, G., Goda, H. A., et al. (2021). Inhibition of *Anabaena* sp. and *Oscillatoria* sp. using probiotic lactic acid bacteria. *Egypt. J. Chem.* 64, 201–210. doi: 10.21608/ejchem.2020.41997.2848

Alamsjah, M. A., Hirao, S., Ishibashi, F., and Fujita, Y. (2005). Isolation and structure determination of algicidal compounds from *Ulva fasciata*. *Biosci. Biotechnol. Biochem.* 69, 2186–2192. doi: 10.1271/bbb.69.2186

Alamsjah, M. A., Hirao, S., Ishibashi, F., Oda, T., and Fujita, Y. (2008). Algicidal activity of polyunsaturated fatty acids derived from *Ulva fasciata* and *U. pertusa* (Ulvaceae, Chlorophyta) on phytoplankton. *J. Appl. Phycol.* 20, 713–720. doi: 10.1007/s10811-007-9257-5

Bagchi, S. N. (1995). Structure and site of action of an algicide from a cyanobacterium, *Oscillatoria late-virens*. *J. Plant Physiol.* 146, 372–374. doi: 10.1016/S0176-1617(11)82072-9

Bazes, A., Silkina, A., Douzenel, P., Faÿ, F., Kervarec, N., Morin, D., et al. (2009). Investigation of the antifouling constituents from the brown alga *Sargassum muticum* (Yendo) Fensholt. *J. Appl. Phycol.* 21, 395–403. doi: 10.1007/s10811-008-9382-9

Bedoshvili, Y., Bayramova, E., Sudakov, N., Klimenkov, I., Kurilkina, M., Likhoshway, Y., et al. (2021). Impact of algicidal *Bacillus mycoides* on diatom *Ulnaria acus* from Lake Baikal. *Diversity* 13, 469. doi: 10.3390/d13100469

Benegas, G. R. S., Bernal, S. P. F., de Oliveira, V. M., and Passarini, M. R. Z. (2021). Antimicrobial activity against *Microcystis aeruginosa* and degradation of microcystin-LR by bacteria isolated from Antarctica. *Environ. Sci. Pollut. Res.* 28, 52381–52391. doi: 10.1007/s11356-021-14458-5

Bhattarai, H. D., Ganti, V. S., Paudel, B., Lee, Y. K., Lee, H. K., Hong, Y.-K., et al. (2007). Isolation of antifouling compounds from the marine bacterium, *Shewanella oneidensis* SCH0402. *World J. Microbiol. Biotechnol.* 23, 243–249. doi: 10.1007/s11274-006-9220-7

Bishop, W. M., Richardson, R. J., and Willis, B. E. (2018). Comparison of partitioning and efficacy between copper algaecide formulations: refining the critical burden concept. *Water. Air. Soil Pollut.* 229, 300. doi: 10.1007/s11270-018-3958-z

Blom, J. F., Brütsch, T., Barbaras, D., Bethuel, Y., Locher, H. H., Hubschwerlen, C., et al. (2006). Potent algicides based on the cyanobacterial alkaloid nostocarboline. *Org. Lett.* 8, 737–740. doi: 10.1021/ol052968b

Cai, G., Yang, X., Lai, Q., Yu, X., Zhang, H., Li, Y., et al. (2016). Lysing bloom-causing alga *Phaeocystis globosa* with microbial algicide: An efficient process that decreases the toxicity of algal exudates. *Sci. Rep.* 6, 20081. doi: 10.1038/srep20081

Cheng, L., He, Y., Tian, Y., Liu, B., Zhang, Y., Zhou, Q., et al. (2017). Comparative biotoxicity of N-Phenyl-1-naphthylamine and N-Phenyl-2-naphthylamine on cyanobacteria *Microcystis aeruginosa*. *Chemosphere* 176, 183–191. doi: 10.1016/j.chemosphere.2017.02.110

Cho, J. Y. (2012a). Algicidal activity of marine *Alteromonas* sp. KNS-16 and isolation of active compounds. *Biosci. Biotechnol. Biochem.* 76, 1452–1458. doi: 10.1271/bbb.120102

Cho, J. Y. (2012b). Antifouling activity of giffinisterone B and oleamide isolated from a filamentous bacterium *Leucothrix mucor* culture against *Ulva pertusa*. *Korean J. Fish. Aquat. Sci.* 45, 30–34. doi: 10.5657/KFAS.2012.0030

Cho, J. Y. (2012c). Antifouling steroids isolated from red alga epiphyte filamentous bacterium *Leucothrix mucor*. *Fish. Sci.* 78, 683–689. doi: 10.1007/s12562-012-0490-8

Cho, J. Y. (2013). Antifouling chromanols isolated from brown alga *Sargassum horneri*. *J. Appl. Phycol.* 25, 299–309. doi: 10.1007/s10811-012-9864-7

Cho, J. Y., and Kim, M. S. (2012). Induction of antifouling diterpene production by *Streptomyces cinnabarinus* PK209 in co-culture with marine-derived *Alteromonas* sp. KNS-16. *Biosci. Biotechnol. Biochem.* 76, 1849–1854. doi: 10.1271/bbb.120221

Cho, S., Cho, S. H., Ko, S.-R., Jeong, Y., Lee, E., Jin, S., et al. (2021). Elucidation of the algicidal mechanism of the marine bacterium *Pseudoruegeria* sp. M32A2M against the harmful alga *Alexandrium catenella* based on time-course transcriptome analysis. *Front. Mar. Sci.* 8, 728890. doi: 10.3389/fmars.2021.728890

Choi, D., Yu, S., Baek, S. H., Kang, Y.-H., Chang, Y.-C., and Cho, H. (2016). Synthesis and algicidal activity of new dichlorobenzylamine derivatives against harmful red tides. *Biotechnol. Bioprocess Eng.* 21, 463–476. doi: 10.1007/s12257-016-0175-8

Churro, C., Alverca, E., Sam-Bento, F., Paulino, S., Figueira, V. C., Bento, A. J., et al. (2009). Effects of bacillamide and newly synthesized derivatives on the growth of cyanobacteria and microalgae cultures. *J. Appl. Phycol.* 21, 429–442. doi: 10.1007/s10811-008-9388-3

DellaGreca, M., Fiorentino, A., Isidori, M., Monaco, P., Temussi, F., and Zarrelli, A. (2001). Antialgal furano-diterpenes from *Potamogeton natans* L. *Phytochemistry* 58, 299–304. doi: 10.1016/S0031-9422(01)00203-5

Ding, N., Du, W., Feng, Y., Song, Y., Wang, C., Li, C., et al. (2021). Algicidal activity of a novel indigenous bacterial strain of *Paracoccus homiensis* against the harmful algal bloom species, *Karenia mikimotoi*. *Arch. Microbiol.* 203, 4821–4828. doi: 10.1007/s00203-021-02468-3

Effiong, K., Hu, J., Xu, C., Zhang, Y., Yu, S., Tang, T., et al. (2022). 3-Indoleacrylic acid from canola straw as a promising antialgal agent - Inhibition effect and mechanism on bloom-forming *Prorocentrum donghaiense*. *Mar. Pollut. Bull.* 178, 113657. doi: 10.1016/j.marpolbul.2022.113657

El Bouaidi, W., Essalhi, S., Douma, M., Tazart, Z., Ounas, A., Enaime, G., et al. (2020). Evaluation of the potentiality of *Vicia faba* and *Opuntia ficus indica* as eco-friendly coagulants to mitigate *Microcystis aeruginosa* blooms. *Desalination Water Treat.* 196, 198–213. doi: 10.5004/dwt.2020.26040

Fei, Y., Yan, W. H., Qin, L. X., Hui, L. Y., Bo, L. X., Hong, Y. L., et al. (2013). Isolation and characterization of an algicidal bacterium indigenous to Lake Taihu with a red pigment able to lyse *Microcystis aeruginosa*. *Biomed. Environ. Sci.* 26, 148–154. doi: 10.3967/0895-3988.2013.02.009

Feng, D. qing, Qiu, Y., Wang, W., Wang, X., Ouyang, P. gang, and Ke, C. huan (2013). Antifouling activities of hymenialdisine and debromohymenialdisine from the sponge *Axinella* sp. *Int. Biodeterior. Biodegrad.* 85, 359–364. doi: 10.1016/j.ibiod.2013.08.014

Gao, Y. N., Liu, B. Y., Xu, D., Zhou, Q. H., Hu, C. Y., Ge, F. J., et al. (2011). Phenolic compounds exuded from two submerged freshwater macrophytes and their allelopathic effects on *Microcystis aeruginosa*. *Pol. J. Environ. Stud.* 20, 1153–1159.

Guo, X., Liu, X., Pan, J., and Yang, H. (2015). Synergistic algicidal effect and mechanism of two diketopiperazines produced by *Chryseobacterium* sp. strain GLY-1106 on the harmful bloom-forming *Microcystis aeruginosa*. *Sci. Rep.* 5, 14720. doi: 10.1038/srep14720

Guo, X., Liu, X., Wu, L., Pan, J., and Yang, H. (2016). The algicidal activity of *Aeromonas* sp. strain GLY-2107 against bloom-forming *Microcystis aeruginosa* is regulated by N-acyl homoserine lactone-mediated quorum sensing. *Environ. Microbiol.* 18, 3867–3883. doi: 10.1111/1462-2920.13346

Gustafsson, S., Hultberg, M., Figueroa, R. I., and Rengefors, K. (2009). On the control of HAB species using low biosurfactant concentrations. *Harmful Algae* 8, 857–863. doi: 10.1016/j.hal.2009.04.002

Hagmann, L., and Jüttner, F. (1996). Fischerellin A, a novel photosystem-II-inhibiting allelochemical of the cyanobacterium *Fischerella muscicola* with antifungal and herbicidal activity. *Tetrahedron Lett.* 37, 6539–6542. doi: 10.1016/0040-4039(96)01445-1

Han, X., Zhu, X., Zhu, S., Wei, L., Hong, Z., Guo, L., et al. (2016). A rational design, synthesis, biological evaluation and structure–activity relationship study of novel inhibitors against cyanobacterial fructose-1,6-bisphosphate aldolase. *J. Chem. Inf. Model.* 56, 73–81. doi: 10.1021/acs.jcim.5b00618

Hao, A., Su, M., Kobayashi, S., Zhao, M., and Iseri, Y. (2022). Multiple roles of bamboo as a regulator of cyanobacterial bloom in aquatic systems. *Sci. Rep.* 12, 1605. doi: 10.1038/s41598-022-05506-2

Hare, C. E., Demir, E., Coyne, K. J., Craig Cary, S., Kirchman, D. L., and Hutchins, D. A. (2005). A bacterium that inhibits the growth of *Pfiesteria piscicida* and other dinoflagellates. *Harmful Algae* 4, 221–234. doi: 10.1016/j.hal.2004.03.001

Harvey, E. L., Deering, R. W., Rowley, D. C., El Gamal, A., Schorn, M., Moore, B. S., et al. (2016). A bacterial quorum-sensing precursor induces mortality in the marine coccolithophore, *Emiliania huxleyi*. *Front. Microbiol.* 7. doi: 10.3389/fmicb.2016.00059

Hirao, S., Tara, K., Kuwano, K., Tanaka, J., and Ishibashi, F. (2012). Algicidal activity of glycerolipids from brown alga *Ishige sinicola* toward red tide microalgae. *Biosci. Biotechnol. Biochem.* 76, 372–374. doi: 10.1271/bbb.110645

Hong, Y., Hu, H. Y., Sakoda, A., and Sagehashi, M. (2010). Isolation and characterization of antialgal allelochemicals from *Arundo donax* L. *Allelopathy J.* 25, 357–368.

Hong, Y. K., and Cho, J. Y. (2013). Effect of seaweed epibiotic bacterium *Streptomyces violaceoruber* SCH-09 on marine fouling organisms. *Fish. Sci.* 79, 469–475. doi: 10.1007/s12562-013-0604-y

Hu, J., Berthold, D. E., Wang, Y., Xiao, X., and Laughinghouse, H. D. (2022). Treatment of the red tide dinoflagellate *Karenia brevis* and brevetoxins using USEPA-registered algaecides. *Harmful Algae* 120, 102347. doi: 10.1016/j.hal.2022.102347

Hu, J., Kokoette, E., Xu, C., Huang, S., Tang, T., Zhang, Y., et al. (2023). Natural algaecide sphingosines identified in hybrid straw decomposition driven by white-rot fungi. *Adv. Sci.* 10, 2300569. doi: 10.1002/advs.202300569

Hu, T., Wang, S., Shan, Y., Zhang, Y., Zhu, Y., and Zheng, L. (2021). Complete genome of marine microalgae associated algicidal bacterium *Sulfitobacter pseudonitzschiae* H46 with quorum sensing system. *Curr. Microbiol.* 78, 3741–3750. doi: 10.1007/s00284-021-02632-4

Huang, S., Zuo, L., Cheng, G., He, Y., Zhang, L., Han, Q., et al. (2023). Design, synthesis and mechanism research of novel algicide based on bioactive fragments synthesis strategy. *Pestic. Biochem. Physiol.* 191, 105344. doi: 10.1016/j.pestbp.2023.105344

Huang, S., Zuo, L., Zhang, L., Guo, X., Cheng, C., He, Y., et al. (2024a). Design, synthesis, and mode of action of thioacetamide derivatives as the algicide candidate based on active substructure splicing strategy. *J. Agric. Food Chem.* 72, 7021–7032. doi: 10.1021/acs.jafc.4c00912

Huang, T., Lai, M., Lin, Z., Luo, R., Xiang, X., Xu, H., et al. (2024b). Identification of algicidal monoterpenoids from four chemotypes of *Cinnamomum* camphora and their algicidal mechanisms on *Microcystis aeruginosa*. *Environ. Res.* 241, 117714. doi: 10.1016/j.envres.2023.117714

Jančula, D., Drábková, M., Černý, J., Karásková, M., Kořínková, R., Rakušan, J., et al. (2008). Algicidal activity of phthalocyanines—Screening of 31 compounds. *Environ. Toxicol.* 23, 218–223. doi: 10.1002/tox.20324

Jeong, S. Y., and Son, H. J. (2021). Effects of mycosubtilin homolog algicides from a marine bacterium, *Bacillus* sp. SY-1, against the harmful algal bloom species *Cochlodinium polykrikoides*. *J. Microbiol.* 59, 389–400. doi: 10.1007/s12275-021-1086-8

Jeong, S.-Y., Ishida, K., Ito, Y., Okada, S., and Murakami, M. (2003). Bacillamide, a novel algicide from the marine bacterium, *Bacillus* sp. SY-1, against the harmful dinoflagellate, *Cochlodinium polykrikoides*. *Tetrahedron Lett.* 44, 8005–8007. doi: 10.1016/j.tetlet.2003.08.115

Jo, D. R., Kim, Y. O., Kim, R., Chang, Y. C., Choi, D., and Cho, H. (2017). Novel rhodanine derivatives are selective algicides against *Microcystis aeruginosa*. *Biotechnol. Bioprocess Eng.* 22, 748–757. doi: 10.1007/s12257-017-0343-5

Kang, P.-G., Hong, J., Kim, E., and Kim, B. (2020). Effects of extracts of reed and cattail on the growth of a cyanobacterium, *Microcystis aeruginosa*. *J. Freshw. Ecol.* Available at: https://www.tandfonline.com/doi/abs/10.1080/02705060.2020.1748128 (Accessed November 19, 2024).

Kim, J. S., Kim, J. C., Lee, S., Lee, B. H., and Cho, K. Y. (2006). Biological activity of l-2-azetidinecarboxylic acid, isolated from *Polygonatum odoratum* var. *pluriflorum*, against several algae. *Aquat. Bot.* 85, 1–6. doi: 10.1016/j.aquabot.2006.01.003

Kim, Y. M., Wu, Y., Duong, T. U., Ghodake, G. S., Kim, S. W., Jin, E., et al. (2010). Thiazolidinediones as a novel class of algicides against red tide harmful algal species. *Appl. Biochem. Biotechnol.* 162, 2273–2283. doi: 10.1007/s12010-010-9001-5

Kim, Y. S., Son, H. J., and Jeong, S. Y. (2015). Isolation of an algicide from a marine bacterium and its effects against the toxic dinoflagellate *Alexandrium catenella* and other harmful algal bloom species. *J. Microbiol.* 53, 511–517. doi: 10.1007/s12275-015-5303-1

Kim, Young Do, Shin, Hyun Woung, and Cho, Ji Young (2013). Antifouling activity of coumarin and its derivatives isolated from the cinnamon tree *Cinnamomum loureiroi*. *Korean J. Fish. Aquat. Sci.* 46, 53–58. doi: 10.5657/KFAS.2013.0053

Ko, S. R., Jeong, Y., Cho, S. H., Lee, E., Jeong, B. S., Baek, S. H., et al. (2022). Functional role of a novel algicidal compound produced by *Pseudoruegeria* sp. M32A2M on the harmful algae *Alexandrium catenella*. *Chemosphere* 300, 134535. doi: 10.1016/j.chemosphere.2022.134535

Kodani, S., Imoto, A., Mitsutani, A., and Murakami, M. (2002). Isolation and identification of the antialgal compound, harmane (1-methyl-β-carboline), produced by the algicidal bacterium, *Pseudomonas* sp. K44-1. *J. Appl. Phycol.* 14, 109–114. doi: 10.1023/A:1019533414018

Kong, C. H., Wang, P., Zhang, C. X., Zhang, M. X., and Hu, F. (2006). Herbicidal potential of allelochemicals from *Lantana camara* against *Eichhornia crassipes* and the alga *Microcystis aeruginosa*. *Weed Res.* 46, 290–295. doi: 10.1111/j.1365-3180.2006.00509.x

Kong, Y., Wang, Q., Chen, Y., Xu, X., Zhu, L., Yao, H., et al. (2020). Anticyanobacterial process and action mechanism of *Streptomyces* sp. HJC-D1 on *Microcystis aeruginosa*. *Environ. Prog. Sustain. Energy* 39, e13392. doi: 10.1002/ep.13392

Le, V. V., Ko, S. R., Kang, M., Lee, S. A., Oh, H. M., and Ahn, C. Y. (2022). Algicide capacity of *Paucibacter aquatile* DH15 on *Microcystis aeruginosa* by attachment and non-attachment effects. *Environ. Pollut.* 302, 119079. doi: 10.1016/j.envpol.2022.119079

Li, D., Kang, X., Chu, L., Wang, Y., Song, X., Zhao, X., et al. (2021). Algicidal mechanism of *Raoultella ornithinolytica* against *Microcystis aeruginosa*: Antioxidant response, photosynthetic system damage and microcystin degradation. *Environ. Pollut.* 287, 117644. doi: 10.1016/j.envpol.2021.117644

Li, D., Zhang, H., Fu, L., An, X., Zhang, B., Li, Y., et al. (2014a). A novel algicide: evidence of the effect of a fatty acid compound from the marine bacterium, *Vibrio* sp. BS02 on the harmful dinoflagellate, *Alexandrium tamarense*. *PLOS ONE* 9, e91201. doi: 10.1371/journal.pone.0091201

Li, F. M., and Hu, H. Y. (2005). Isolation and characterization of a novel antialgal allelochemical from *phragmites communis*. *Appl. Environ. Microbiol.* 71, 6545–6553. doi: 10.1128/AEM.71.11.6545-6553.2005

Li, H., Ai, H., Kang, L., Sun, X., and He, Q. (2016). Simultaneous *Microcystis* algicidal and microcystin degrading capability by a single *Acinetobacter* bacterial strain. *Environ. Sci. Technol.* 50, 11903–11911. doi: 10.1021/acs.est.6b03986

Li, Z., Geng, M., and Yang, H. (2015). Algicidal activity of *Bacillus* sp. Lzh-5 and its algicidal compounds against *Microcystis aeruginosa*. *Appl. Microbiol. Biotechnol.* 99, 981–990. doi: 10.1007/s00253-014-6043-6

Li, Z., Lin, S., Liu, X., Tan, J., Pan, J., and Yang, H. (2014b). A freshwater bacterial strain, *Shewanella* sp. Lzh-2, isolated from Lake Taihu and its two algicidal active substances, hexahydropyrrolo[1,2-a]pyrazine-1,4-dione and 2, 3-indolinedione. *Appl. Microbiol. Biotechnol.* 98, 4737–4748. doi: 10.1007/s00253-014-5602-1

Liang, D., Xiang, H., and Xia, J. (2022). Inhibitory effects of *Ipomoea cairica* extracts on the harmful algae *Phaeocystis globosa*. *Mar. Pollut. Bull.* 185, 114228. doi: 10.1016/j.marpolbul.2022.114228

Lin, S., Geng, M., Liu, X., Tan, J., and Yang, H. (2016). On the control of *Microcystis aeruginosa* and *Synechococccus* species using an algicidal bacterium, *Stenotrophomonas* F6, and its algicidal compounds cyclo-(Gly-Pro) and hydroquinone. *J. Appl. Phycol.* 28, 345–355. doi: 10.1007/s10811-015-0549-x

Liu, F., Feng, S., Ali Nasser Mansoor Al-Haimi, A., Zhu, S., Chen, H., Feng, P., et al. (2024). Discovery of two novel bioactive algicidal substances from *Brevibacillus* sp. via metabolomics profiling and back-validation. *J. Hazard. Mater.* 469, 133985. doi: 10.1016/j.jhazmat.2024.133985

Liu, Y.-M., Chen, M.-J., Wang, M.-H., Jia, R.-B., and Li, L. (2013). Inhibition of *Microcystis aeruginosa* by the extracellular substances from an *Aeromonas* sp. 23, 1304–1307. doi: 10.4014/jmb.1304.04025

Liu, Z. Z., Zhu, J. P., Li, M., Xue, Q. Q., Zeng, Y., and Wang, Z. P. (2014). Effects of freshwater bacterial siderophore on *Microcystis* and *Anabaena*. *Biol. Control* 78, 42–48. doi: 10.1016/j.biocontrol.2014.07.010

Lu, H., Xie, H., Gong, Y., Wang, Q., and Yang, Y. (2011). Secondary metabolites from the seaweed *Gracilaria lemaneiformis* and their allelopathic effects on *Skeletonema costatum*. *Biochem. Syst. Ecol.* 39, 397–400. doi: 10.1016/j.bse.2011.05.015

Lu, L., Niu, X., Zhang, D., Ma, J., Zheng, X., Xiao, H., et al. (2021). The algicidal efficacy and the mechanism of *Enterobacter* sp. EA-1 on *Oscillatoria* dominating in aquaculture system. *Environ. Res.* 197, 111105. doi: 10.1016/j.envres.2021.111105

Lu, X., Zhou, B., Xu, L., Liu, L., Wang, G., Liu, X., et al. (2016). A marine algicidal *Thalassospira* and its active substance against the harmful algal bloom species *Karenia mikimotoi*. *Appl. Microbiol. Biotechnol.* 100, 5131–5139. doi: 10.1007/s00253-016-7352-8

Luo, J., Wang, Y., Tang, S., Liang, J., Lin, W., and Luo, L. (2013). Isolation and identification of algicidal compound from streptomyces and algicidal mechanism to *Microcystis aeruginosa*. *PLOS ONE* 8, e76444. doi: 10.1371/journal.pone.0076444

Luo, Y., Yang, Y., Hou, W., and Fu, J. (2021). Novel algicides against bloom-forming cyanobacteria from allelochemicals: design, synthesis, bioassay, and 3D-QSAR study. *Biology* 10, 1145. doi: 10.3390/biology10111145

Mary, M. A., Tábora-Sarmiento, S., Nash, S., Mayer, G. D., Crago, J., and Patiño, R. (2024). Plant-derived products selectively suppress growth of the harmful alga *Prymnesium parvum*. *Water* 16, 930. doi: 10.3390/w16070930

Meepagala, K. M., Schrader, K. K., Wedge, D. E., and Duke, S. O. (2005). Algicidal and antifungal compounds from the roots of *Ruta graveolens* and synthesis of their analogs. *Phytochemistry* 66, 2689–2695. doi: 10.1016/j.phytochem.2005.09.019

Mizuno, C. S., Schrader, K. K., and Rimando, A. M. (2008). Algicidal activity of stilbene analogues. *J. Agric. Food Chem.* 56, 9140–9145. doi: 10.1021/jf801988p

Mohamed, Z., Alamri, S., and Hashem, M. (2022). Simultaneous biodegradation of harmful *Cylindrospermopsis raciborskii* and cylindrospermopsin toxin in batch culture by single *Bacillus* strain. *Environ. Sci. Pollut. Res.* 29, 5153–5161. doi: 10.1007/s11356-021-16062-z

Murray, D., Jefferson, B., Jarvis, P., and Parsons, S. A. (2010). Inhibition of three algae species using chemicals released from barley straw. *Environ. Technol.* 31, 455–466. doi: 10.1080/09593331003663294

Nakai, S., Yamada, S., and Hosomi, M. (2005). Anti-cyanobacterial fatty acids released from *Myriophyllum spicatum*. *Hydrobiologia* 543, 71–78. doi: 10.1007/s10750-004-6822-7

Nakai, S., Zhou, S., Masaaki Hosomi, and Hosomi M. (n.d.). Allelopathic growth inhibition of cyanobacteria by reed. *Allelopathy J.* Available at: https://www.indianjournals.com/ijor.aspx?target=ijor:aj&volume=18&issue=2&article=008&type=fulltext (Accessed November 26, 2024).

Nakashima, T., Miyazaki, Y., Matsuyama, Y., Muraoka, W., Yamaguchi, K., and Oda, T. (2006). Producing mechanism of an algicidal compound against red tide phytoplankton in a marine bacterium γ-proteobacterium. *Appl. Microbiol. Biotechnol.* 73, 684–690. doi: 10.1007/s00253-006-0507-2

Nanayakkara, N. P. D., and Schrader, K. K. (2008). Synthesis of water-soluble 9,10-anthraquinone analogues with potent cyanobactericidal activity toward the musty-odor cyanobacterium *Oscillatoria perornata*. *J. Agric. Food Chem.* 56, 1002–1007. doi: 10.1021/jf072836s

Ouyang, L., Liu, Y., Chen, H., Zaynab, M., Yang, X., Wang, S., et al. (2021). Encapsulation and algicidal properties of fermentation products from *Vibrio brasiliensis* H115. *Front. Mar. Sci.* 8. doi: 10.3389/fmars.2021.676913

Ozaki, K., Ohta, A., Iwata, C., Horikawa, A., Tsuji, K., Ito, E., et al. (2008). Lysis of cyanobacteria with volatile organic compounds. *Chemosphere* 71, 1531–1538. doi: 10.1016/j.chemosphere.2007.11.052

Park, M. H., Chung, I. M., Ahmad, A., Kim, B. H., and Hwang, S. J. (2009). Growth inhibition of unicellular and colonial *Microcystis* strains (*Cyanophyceae*) by compounds isolated from rice (*Oryza sativa*) hulls. *Aquat. Bot.* 90, 309–314. doi: 10.1016/j.aquabot.2008.11.007

Park, M. H., Han, M. S., Ahn, C. Y., Kim, H. S., Yoon, B. D., and Oh, H. M. (2006). Growth inhibition of bloom‐forming cyanobacterium *Microcystis aeruginosa* by rice straw extract. *Lett. Appl. Microbiol.* 43, 307–312. doi: 10.1111/j.1472-765X.2006.01951.x

Park, M. H., Kim, K., and Hwang, S. J. (2020). Differential effects of the allelochemical juglone on growth of harmful and non-target freshwater algae. *Appl. Sci.* 10, 2873. doi: 10.3390/app10082873

Pei, Y., Liu, L., Hilt, S., Xu, R., Wang, B., Li, C., et al. (2018). Root exudated algicide of *Eichhornia crassipes* enhances allelopathic effects of cyanobacteria *Microcystis aeruginosa* on green algae. *Hydrobiologia* 823, 67–77. doi: 10.1007/s10750-018-3696-7

Pokrzywinski, K. L., Place, A. R., Warner, M. E., and Coyne, K. J. (2012). Investigation of the algicidal exudate produced by *Shewanella* sp. IRI-160 and its effect on dinoflagellates. *Harmful Algae* 19, 23–29. doi: 10.1016/j.hal.2012.05.002

Purcaro, R., Schrader, K. K., Burandt, C., DellaGreca, M., and Meepagala, K. M. (2009). Algicide constituents from Swinglea glutinosa. *J. Agric. Food Chem.* 57, 10632–10635. doi: 10.1021/jf902561c

Qi, J., Song, Y., Liang, J., Bai, Y., Hu, C., Liu, H., et al. (2021). Growth inhibition of *Microcystis aeruginosa* by sand-filter prevalent manganese-oxidizing bacterium. *Sep. Purif. Technol.* 256, 117808. doi: 10.1016/j.seppur.2020.117808

Quan, H., Zhang, Y., Yin, P., and Zhao, L. (2021). Effects of two algicidal substances, *ortho*-tyrosine and urocanic acid, on the growth and physiology of *Heterosoigma akashiwo*. *Environ. Pollut.* 284, 117004. doi: 10.1016/j.envpol.2021.117004

Rose, M. M., Scheer, D., Hou, Y., Hotter, V. S., Komor, A. J., Aiyar, P., et al. (2021). The bacterium *Pseudomonas protegens* antagonizes the microalga *Chlamydomonas reinhardtii* using a blend of toxins. *Environ. Microbiol.* 23, 5525–5540. doi: 10.1111/1462-2920.15700

Sang, W., Du, C., Ni, L., Li, S., Hamad, A. A. A., Xu, C., et al. (2024). Physiological and molecular mechanisms of the inhibitory effects of artemisinin on *Microcystis aeruginosa* and *Chlorella pyrenoidosa*. *J. Hazard. Mater.* 470, 134241. doi: 10.1016/j.jhazmat.2024.134241

Schrader, K. K. (2005). Evaluation of several commercial algicides for control of odor-producing cyanobacteria. *J. Aquat. Plant Manag.* 43, 100–102.

Schrader, K. K., Bommer, J. C., and Jori, G. (2010). In vitro evaluation of the antimicrobial agent AquaFrin as a bactericide and selective algicide for use in channel catfish aquaculture. *North Am. J. Aquac.* 72, 304–308. doi: 10.1577/A09-076.1

Schrader, K. K., Nanayakkara, N. P. D., Tucker, C. S., Rimando, A. M., Ganzera, M., and Schaneberg, B. T. (2003). Novel derivatives of 9,10-anthraquinone are selective algicides against the musty-odor cyanobacterium *Oscillatoria perornata*. *Appl. Environ. Microbiol.* 69, 5319–5327. doi: 10.1128/AEM.69.9.5319-5327.2003

Shao, J., Liu, D., Gong, D., Zeng, Q., Yan, Z., and Gu, J.-D. (2013). Inhibitory effects of sanguinarine against the cyanobacterium *Microcystis aeruginosa* NIES-843 and possible mechanisms of action. *Aquat. Toxicol.* 142–143, 257–263. doi: 10.1016/j.aquatox.2013.08.019

Sun, X. X., Choi, J. K., and Kim, E. K. (2004). A preliminary study on the mechanism of harmful algal bloom mitigation by use of sophorolipid treatment. *J. Exp. Mar. Biol. Ecol.* 304, 35–49. doi: 10.1016/j.jembe.2003.11.020

Sun, Y., Meng, K., Su, Z., Guo, G., Pu, Y., and Wang, C. (2017). Isolation and purification of antialgal compounds from the red alga *Gracilaria lemaneiformis* for activity against common harmful red tide microalgae. *Environ. Sci. Pollut. Res.* 24, 4964–4972. doi: 10.1007/s11356-016-8256-y

Sun, Y., Zhou, J., Han, X., Zhang, N., Yang, Z., and Zhang, X. (2021). Several natural products isolated from a red alga *Gracilaria lemaneiformis* and its evaluation of antialgal activity against six common red tide microalgae. *Environ. Sci. Pollut. Res.* 28, 22409–22426. doi: 10.1007/s11356-020-11755-3

Tebaa, L., Douma, M., Tazart, Z., Mouhri, K., and Loudiki, M. (2021). Control of *Microcystis aeruginosa* toxic blooms by Moroccan medicinal plant-based algicides. *Desalination Water Treat.* 237, 146–158. doi: 10.5004/dwt.2021.27726

Umetsu, S., Kanda, M., Imai, I., Sakai, R., and Fujita, M. J. (2019). Questiomycins, algicidal compounds produced by the marine bacterium *Alteromonas* sp. D and their production cue. *Molecules* 24, 4522. doi: 10.3390/molecules24244522

Wang, B., Tao, Y., Liu, Q., Liu, N., Jin, Z., and Xu, X. (2017). Algicidal activity of bacillamide alkaloids and their analogues against marine and freshwater harmful algae. *Mar. Drugs* 15, 247. doi: 10.3390/md15080247

Wang, B., Yang, X., Lu, J., Zhou, Y., Su, J., Tian, Y., et al. (2012). A marine bacterium producing protein with algicidal activity against *Alexandrium tamarense*. *Harmful Algae* 13, 83–88. doi: 10.1016/j.hal.2011.10.006

Wang, H., Zhu, H., Zhang, L., Xue, W., and Yuan, B. (2014). Identification of antialgal compounds from the aquatic plant *Elodea nuttallii*. *Allelopathy J.* 34, 207–213.

Wang, M. H., Peng, P., Liu, Y. M., Jia, R. B., and Li, L. (2013). Algicidal activity of a dibenzofuran-degrader *Rhodococcus* sp. *J. Microbiol. Biotechnol.* 23, 260–266. doi: 10.4014/jmb.1208.08018

Wang, R., and Seyedsayamdost, M. R. (2017). Roseochelin B, an algaecidal natural product synthesized by the *Roseobacter Phaeobacter inhibens* in response to algal sinapic acid. *Org. Lett.* 19, 5138–5141. doi: 10.1021/acs.orglett.7b02424

Wang, S., Yang, S., Zuo, J., Hu, C., Song, L., Gan, N., et al. (2021). Simultaneous removal of the freshwater bloom-forming cyanobacterium *Microcystis* and cyanotoxin microcystins via combined use of algicidal bacterial filtrate and the microcystin-degrading enzymatic agent, MlrA. *Microorganisms* 9, 1594. doi: 10.3390/microorganisms9081594

Wang, X., Gong, L., Liang, S., Han, X., Zhu, C., and Li, Y. (2005). Algicidal activity of rhamnolipid biosurfactants produced by *Pseudomonas aeruginosa*. *Harmful Algae* 4, 433–443. doi: 10.1016/j.hal.2004.06.001

Wang, Y., Li, S., Liu, G., Li, X., Yang, Q., Xu, Y., et al. (2020). Continuous production of algicidal compounds against *Akashiwo sanguinea* via a *Vibrio* sp. co-culture. *Bioresour. Technol.* 295, 122246. doi: 10.1016/j.biortech.2019.122246

Wang, Y., Liu, Q., Wei, Z., Liu, N., Li, Y., Li, D., et al. (2018). Thiazole amides, a novel class of algaecides against freshwater harmful algae. *Sci. Rep.* 8, 8555. doi: 10.1038/s41598-018-26911-6

Weiss, G., Kovalerchick, D., Lieman-Hurwitz, J., Murik, O., De Philippis, R., Carmeli, S., et al. (2019). Increased algicidal activity of *Aeromonas veronii* in response to *Microcystis aeruginosa*: interspecies crosstalk and secondary metabolites synergism. *Environ. Microbiol.* 21, 1140–1150. doi: 10.1111/1462-2920.14561

Whalen, K. E., Kirby, C., Nicholson, R. M., O’Reilly, M., Moore, B. S., and Harvey, E. L. (2018). The chemical cue tetrabromopyrrole induces rapid cellular stress and mortality in phytoplankton. *Sci. Rep.* 8, 15498. doi: 10.1038/s41598-018-33945-3

Wong, B. Y. K., Chen, Y. H., Cui, K. H., Zhou, H. C., Li, F. L., Tam, N. F.-Y., et al. (2024). Differential allelopathic effects of mangrove plants *Kandelia obovata* and *Aegiceras corniculatum* on harmful algal species: Potential applications in algal bloom control. *Mar. Pollut. Bull.* 207, 116874. doi: 10.1016/j.marpolbul.2024.116874

Wu, L., Guo, X., Liu, X., and Yang, H. (2017). NprR-NprX quorum-sensing system regulates the algicidal activity of *Bacillus* sp. strain S51107 against bloom-forming cyanobacterium *Microcystis aeruginosa*. *Front. Microbiol.* 8. doi: 10.3389/fmicb.2017.01968

Wu, Y., Liu, J., Yang, L., Chen, H., Zhang, S., Zhao, H., et al. (2011). Allelopathic control of cyanobacterial blooms by periphyton biofilms. *Environ. Microbiol.* 13, 604–615.

Xiao, S., Wei, L., Hong, Z., Rao, L., Ren, Y., Wan, J., et al. (2019). Design, synthesis and algicides activities of thiourea derivatives as the novel scaffold aldolase inhibitors. *Bioorg. Med. Chem.* 27, 805–812. doi: 10.1016/j.bmc.2019.01.023

Xiao, X., Huang, H., Ge, Z., Rounge, T. B., Shi, J., Xu, X., et al. (2014). A pair of chiral flavonolignans as novel anti-cyanobacterial allelochemicals derived from barley straw (Hordeum vulgare): characterization and comparison of their anti-cyanobacterial activities. *Environ. Microbiol.* 16, 1238–1251. doi: 10.1111/1462-2920.12226

Xu, C., Yu, S., Hu, J., Effiong, K., Ge, Z., Tang, T., et al. (2022). Programmed cell death process in freshwater *Microcystis aeruginosa* and marine *Phaeocystis globosa* induced by a plant derived allelochemical. *Sci. Total Environ.* 838, 156055. doi: 10.1016/j.scitotenv.2022.156055

Yang, J., Zhu, Q., Chai, J., Xu, F., Ding, Y., Zhu, Q., et al. (2022). Development of environmentally friendly biological algicide and biochemical analysis of inhibitory effect of diatom *Skeletonema costatum*. *Chin. Chem. Lett.* 33, 1358–1364. doi: 10.1016/j.cclet.2021.09.053

Yi, Y. L., Lei, Y., Yin, Y. B., Zhang, H. Y., and Wang, G. X. (2012). The antialgal activity of 40 medicinal plants against *Microcystis aeruginosa*. *J. Appl. Phycol.* 24, 847–856. doi: 10.1007/s10811-011-9703-2

Yoshikawa, K., Adachi, K., Nishijima, M., Takadera, T., Tamaki, S., Harada, K., et al. (2000). β-cyanoalanine production by marine bacteria on cyanide-free medium and its specific inhibitory activity toward cyanobacteria. *Appl. Environ. Microbiol.* 66, 718–722. doi: 10.1128/AEM.66.2.718-722.2000

Yu, X., Cai, G., Wang, H., Hu, Z., Zheng, W., Lei, X., et al. (2018). Fast-growing algicidal *Streptomyces* sp. U3 and its potential in harmful algal bloom controls. *J. Hazard. Mater.* 341, 138–149. doi: 10.1016/j.jhazmat.2017.06.046

Zeng, Y., Wang, J., Yang, C., Ding, M., Hamilton, P. B., Zhang, X., et al. (2021). A *Streptomyces globisporus* strain kills *Microcystis aeruginosa* via cell-to-cell contact. *Sci. Total Environ.* 769, 144489. doi: 10.1016/j.scitotenv.2020.144489

Zhang, B. H., Ding, Z.-G., Li, H. Q., Mou, X., Zhang, Y. Q., Yang, J. Y., et al. (2016). Algicidal activity of *Streptomyces eurocidicus* JXJ-0089 metabolites and their effects on *Microcystis* physiology. *Appl. Environ. Microbiol.* 82, 5132–5143. doi: 10.1128/AEM.01198-16

Zhang, B., Yang, Y., Xie, W., He, W., Xie, J., and Liu, W. (2022). Identifying algicides of *Enterobacter hormaechei* F2 for control of the harmful Alga *Microcystis aeruginosa*. *Int. J. Environ. Res. Public. Health* 19, 7556. doi: 10.3390/ijerph19137556

Zhang, C., Yi, Y. L., Hao, K., Liu, G. L., and Wang, G. X. (2013). Algicidal activity of *Salvia miltiorrhiza* Bung on *Microcystis aeruginosa*—Towards identification of algicidal substance and determination of inhibition mechanism. *Chemosphere* 93, 997–1004. doi: 10.1016/j.chemosphere.2013.05.068

Zhang, F., Fan, Y., Zhang, D., Chen, S., Bai, X., Ma, X., et al. (2020a). Effect and mechanism of the algicidal bacterium *Sulfitobacter porphyrae* ZFX1 on the mitigation of harmful algal blooms caused by *Prorocentrum donghaiense*. *Environ. Pollut.* 263, 114475. doi: 10.1016/j.envpol.2020.114475

Zhang, H., Wang, H., Zheng, W., Yao, Z., Peng, Y., Zhang, S., et al. (2017). Toxic effects of prodigiosin secreted by *Hahella* sp. KA22 on harmful alga *Phaeocystis globosa*. *Front. Microbiol.* 8. doi: 10.3389/fmicb.2017.00999

Zhang, S., Han, B., Wu, F., and Huang, H. (2020b). Quantitative proteomic analysis provides insights into the algicidal mechanism of *Halobacillus* sp. P1 against the marine diatom *Skeletonema costatum*. *Sci. Total Environ.* 717, 137048. doi: 10.1016/j.scitotenv.2020.137048

Zhang, Z., Li, D., Xie, R., Guo, R., Nair, S., Han, H., et al. (2023). Plastoquinone synthesis inhibition by tetrabromo biphenyldiol as a widespread algicidal mechanism of marine bacteria. *ISME J.*, 1–14. doi: 10.1038/s41396-023-01510-0

Zhao, G., Hong, Y., Li, L., Zhang, H., Xu, R., and Hao, Y. (2022). Selection and characterization of plant-derived alkaloids with strong antialgal inhibition: growth inhibition selectivity and inhibitory mechanism. *Harmful Algae* 117, 102272. doi: 10.1016/j.hal.2022.102272

Zhao, L., Chen, L., and Yin, P. (2014). Algicidal metabolites produced by *Bacillus* sp. strain B1 against *Phaeocystis globosa*. *J. Ind. Microbiol. Biotechnol.* 41, 593–599. doi: 10.1007/s10295-013-1393-0

Zheng, X., Zhang, B., Zhang, J., Huang, L., Lin, J., Li, X., et al. (2013). A marine algicidal actinomycete and its active substance against the harmful algal bloom species *Phaeocystis globosa*. *Appl. Microbiol. Biotechnol.* 97, 9207–9215. doi: 10.1007/s00253-012-4617-8

Zhou, L. H., Zheng, T. L., Chen, X. H., Wang, X., Chen, S. B., Tian, Y., et al. (2008). The inhibitory effects of garlic (*Allium sativum*) and diallyl trisulfide on *Alexandrium tamarense* and other harmful algal species. *J. Appl. Phycol.* 20, 349–358. doi: 10.1007/s10811-007-9262-8

Zhou, L. H., Zheng, T. L., Wang, X., Ye, J. L., Tian, Y., and Hong, H. S. (2007). Effect of five chinese traditional medicines on the biological activity of a red-tide causing alga—*Alexandrium tamarense*. *Harmful Algae* 6, 354–360. doi: 10.1016/j.hal.2006.10.002

Zhou, Q., Zhang, Y., Han, S., Wang, Y., Qin, H., and Zhang, Z. (2021). Physiological responses of *Microcystis aeruginosa* to extracellular degradative enzymes and algicidal substance from heterotrophic bacteria. *Pol. J. Environ. Stud.* 30, 2947–2955. doi: 10.15244/pjoes/127867

Zhu, J., Liu, B., Wang, J., Gao, Y., and Wu, Z. (2010). Study on the mechanism of allelopathic influence on cyanobacteria and chlorophytes by submerged macrophyte (*Myriophyllum spicatum*) and its secretion. *Aquat. Toxicol.* 98, 196–203. doi: 10.1016/j.aquatox.2010.02.011

Zhuang, L., Zhao, L., and Yin, P. (2018). Combined algicidal effect of urocanic acid, *N*-acetylhistamine and _L_-histidine to harmful alga *Phaeocystis globosa*. *RSC Adv.* 8, 12760–12766. doi: 10.1039/C8RA00749G
